# Supplementary material for: Tetrahydropyridines’ Stereoselective Formation, How Lockdown Assisted in the Identification of the Features of Its Mechanism
Source: Molecules. 2022 Jul 7;27(14):4367. doi: 10.3390/molecules27144367 (PMC9324243; doi:10.3390/molecules27144367)
Supplement: Supplementary file 1 [file molecules-27-04367-s001.zip › molecules-1750023-supplementary.pdf]

# Stereoselective Multicomponent Synthesis and Mechanism Features of 1,4,5,6-Tetrahydropyridines Formation

Taigib M. Iliyasov, Kirill A. Karpenko, Radmir N. Akchurin, Mikhail E. Minyaev, Anatoly N. Vereshchagin*

N. D. Zelinsky Institute of Organic Chemistry, Russian Academy of Sciences, 47 Leninsky Prosp., Moscow 119991, Russian Federation

vereshchagin@ioc.ac.ru

# Supplementary Materials

## Table of contents

$^{1}H$ and $^{13}C$ NMR spectra of novel compounds **4-8**..........2

2D spectra of compounds **6d**, **7** ..........29

DFT calculations ..........33

## $^{1}H$ and $^{13}C$ NMR spectra of novel compounds 4-8

$^{1}H$ NMR of methyl (4*SR*, 6*RS*)-5,5-dicyano-2-methyl-4,6-bis(2-methyl)phenyl-1,4,5,6-tetrahydropyridine-3-carboxylate (**4b**)

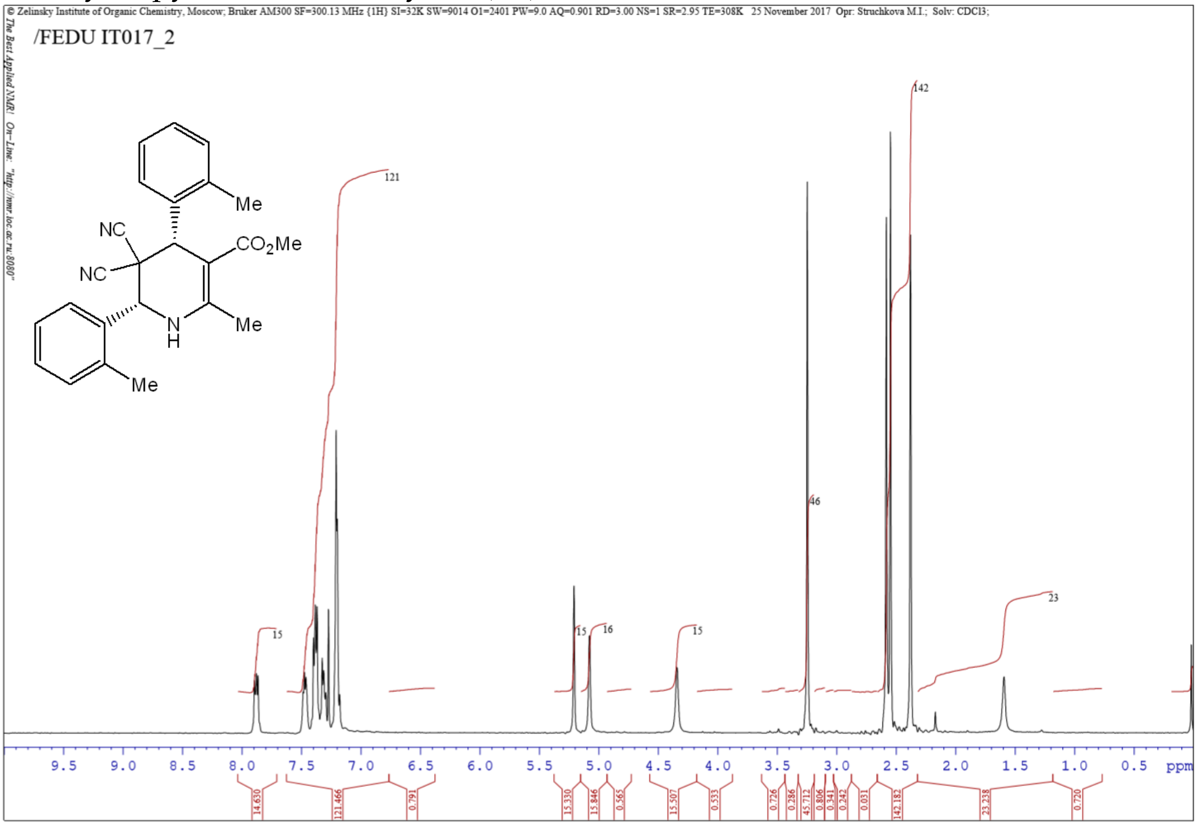

$^{13}C$ NMR of **4b**

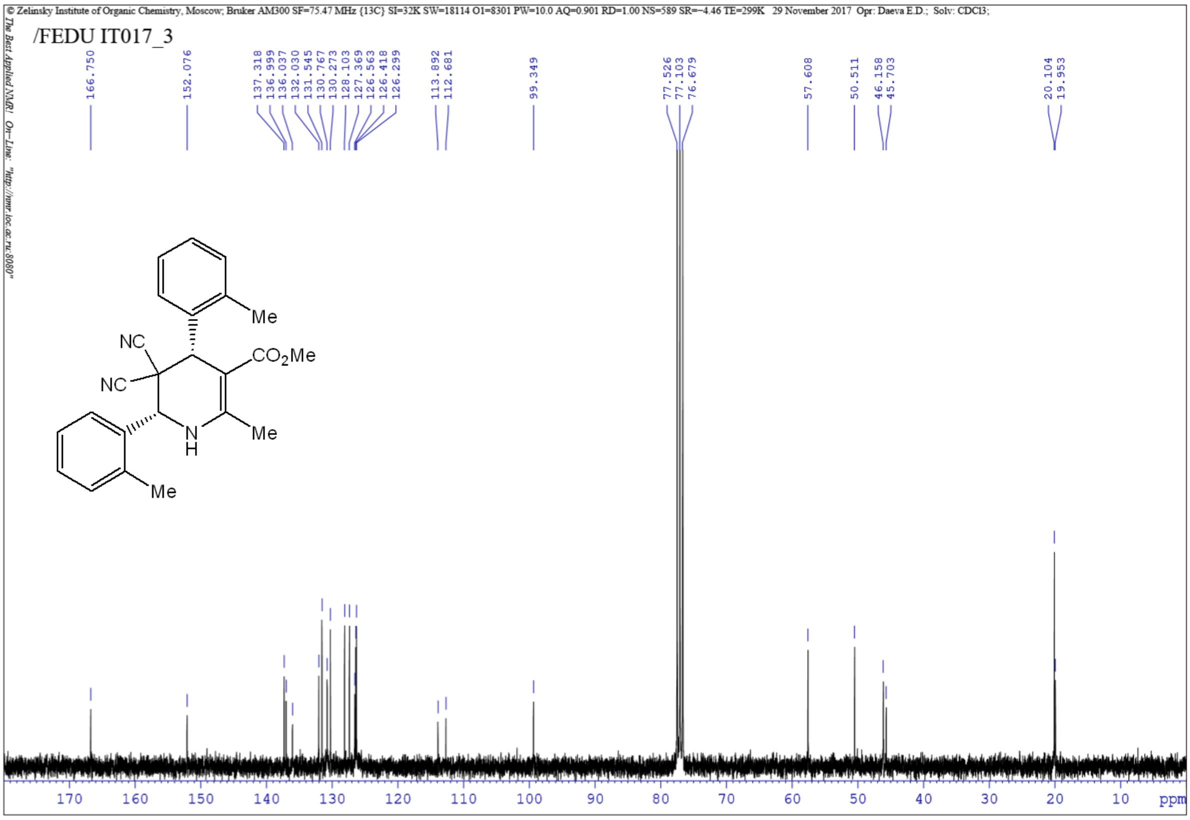

$^{1}$H NMR of methyl (4*SR*, 6*RS*)-5,5-dicyano-2-methyl-4,6-bis(3-methyl)phenyl-1,4,5,6-tetrahydropyridine-3-carboxylate (**4c**)

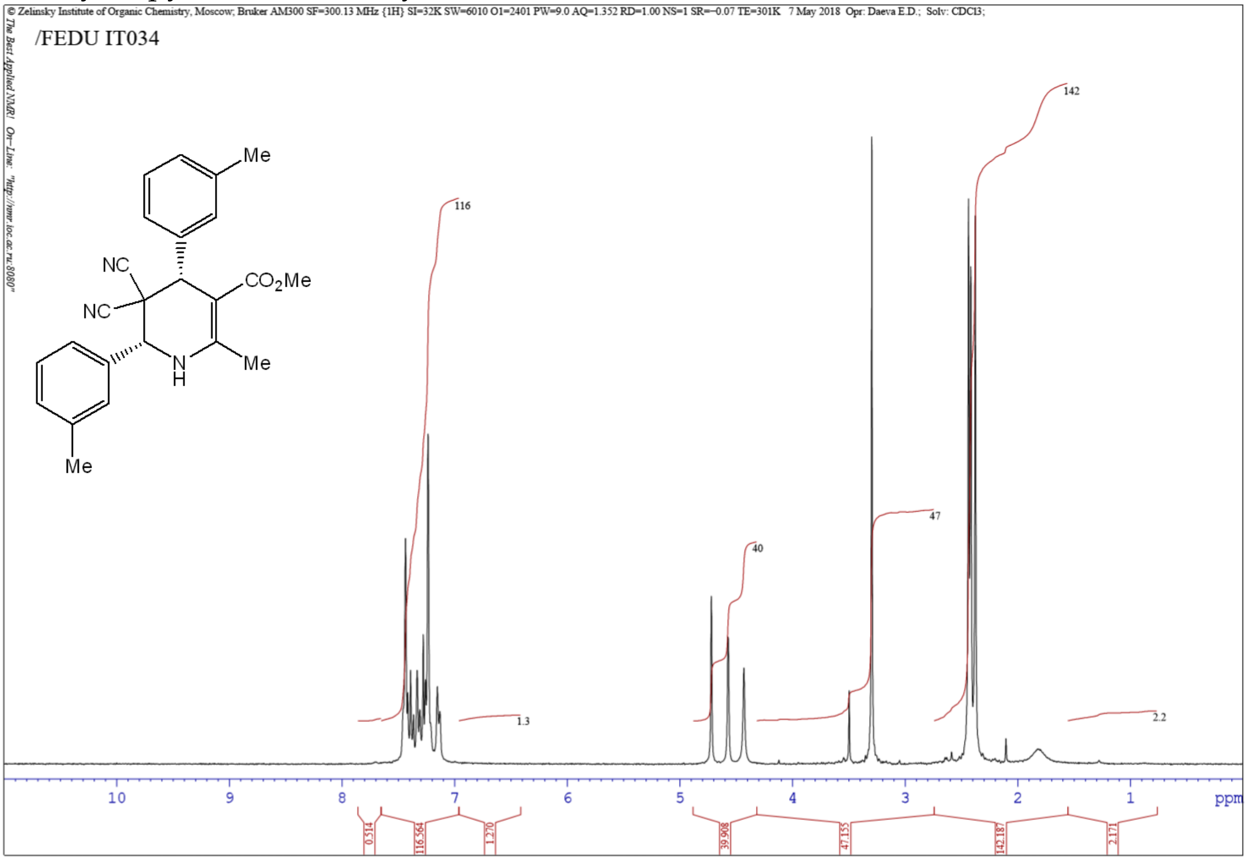


$^{13}$C NMR of **4c**

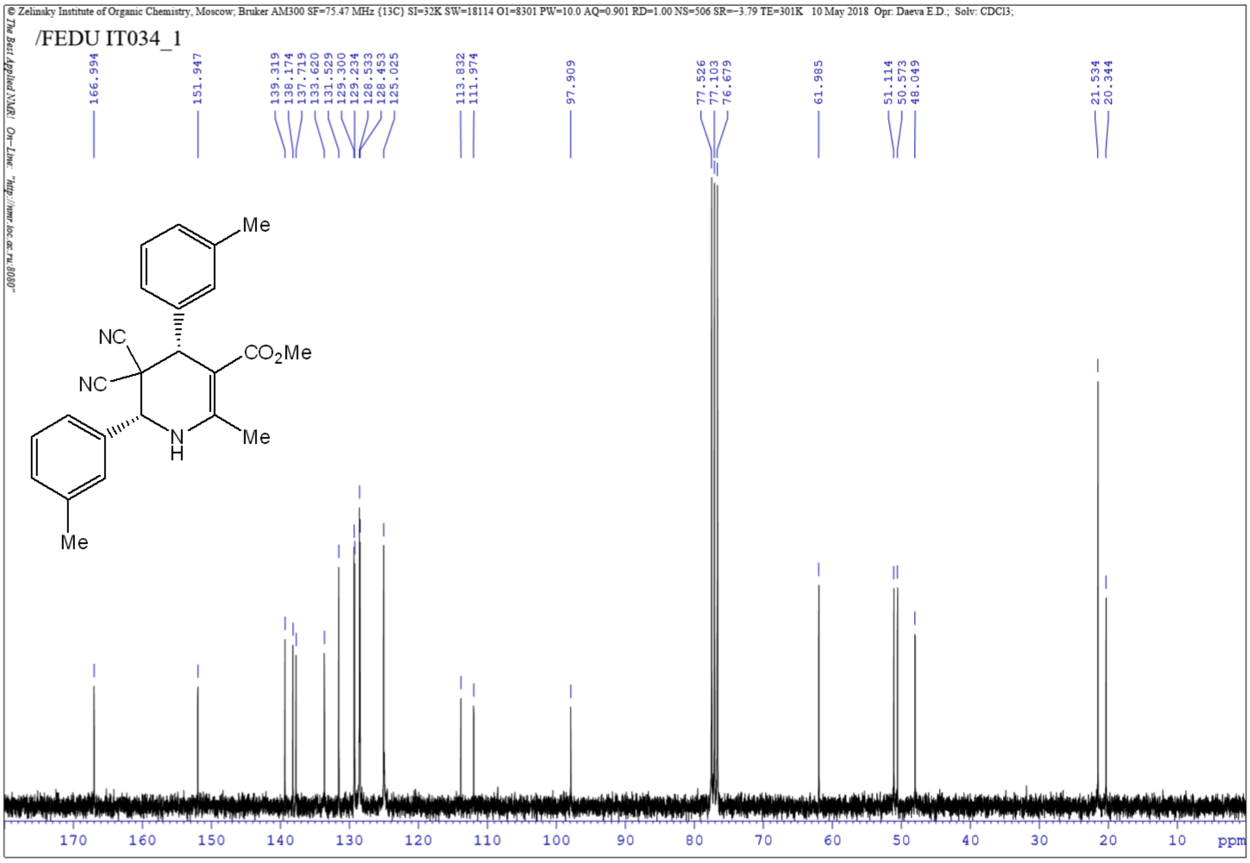

$^{1}$H NMR of methyl (4*SR*, 6*RS*)-5,5-dicyano-2-methyl-4,6-bis(4-methyl)phenyl-1,4,5,6-tetrahydropyridine-3-carboxylate (**4d**)

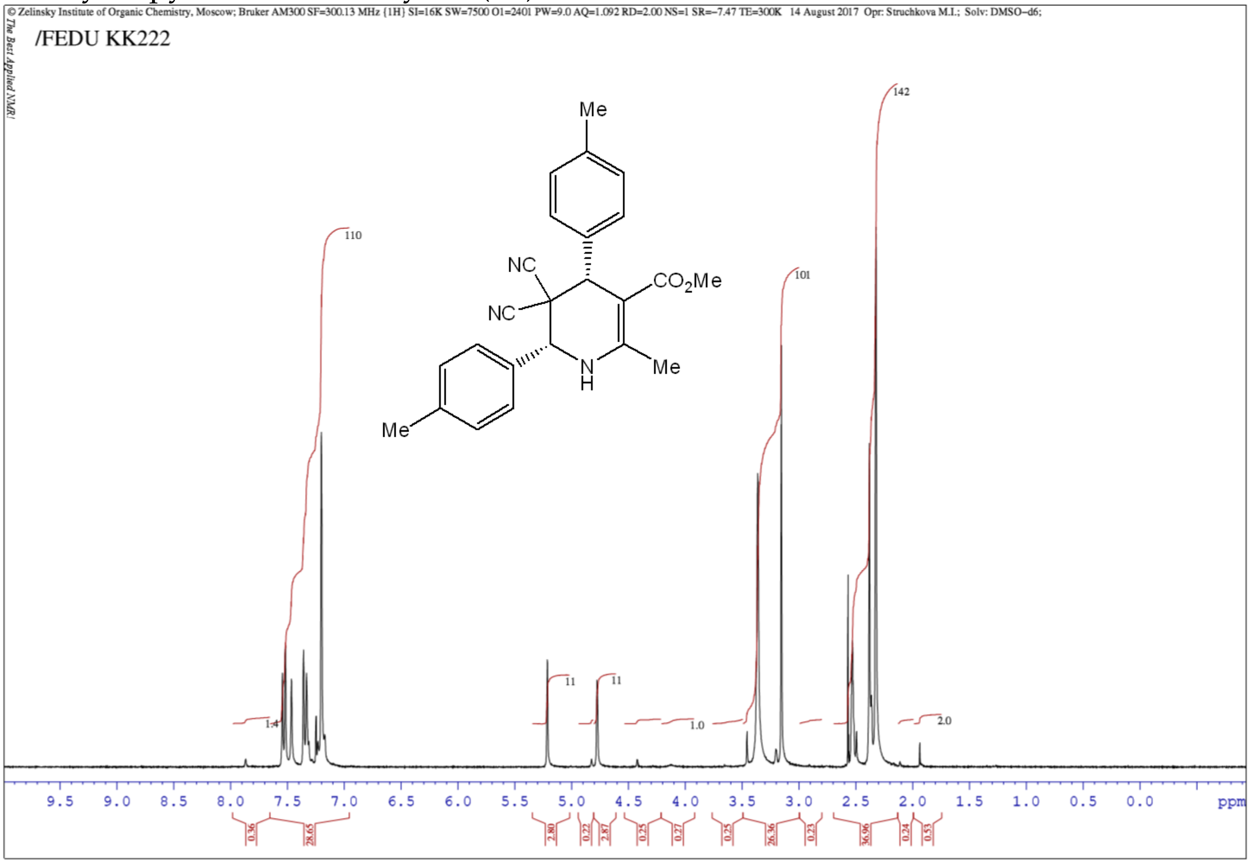

$^{1}H$ NMR of methyl (4*SR*, 6*RS*)-5,5-dicyano-2-methyl-4,6-bis(3-fluoro)phenyl-1,4,5,6-tetrahydropyridine-3-carboxylate (**4e**)

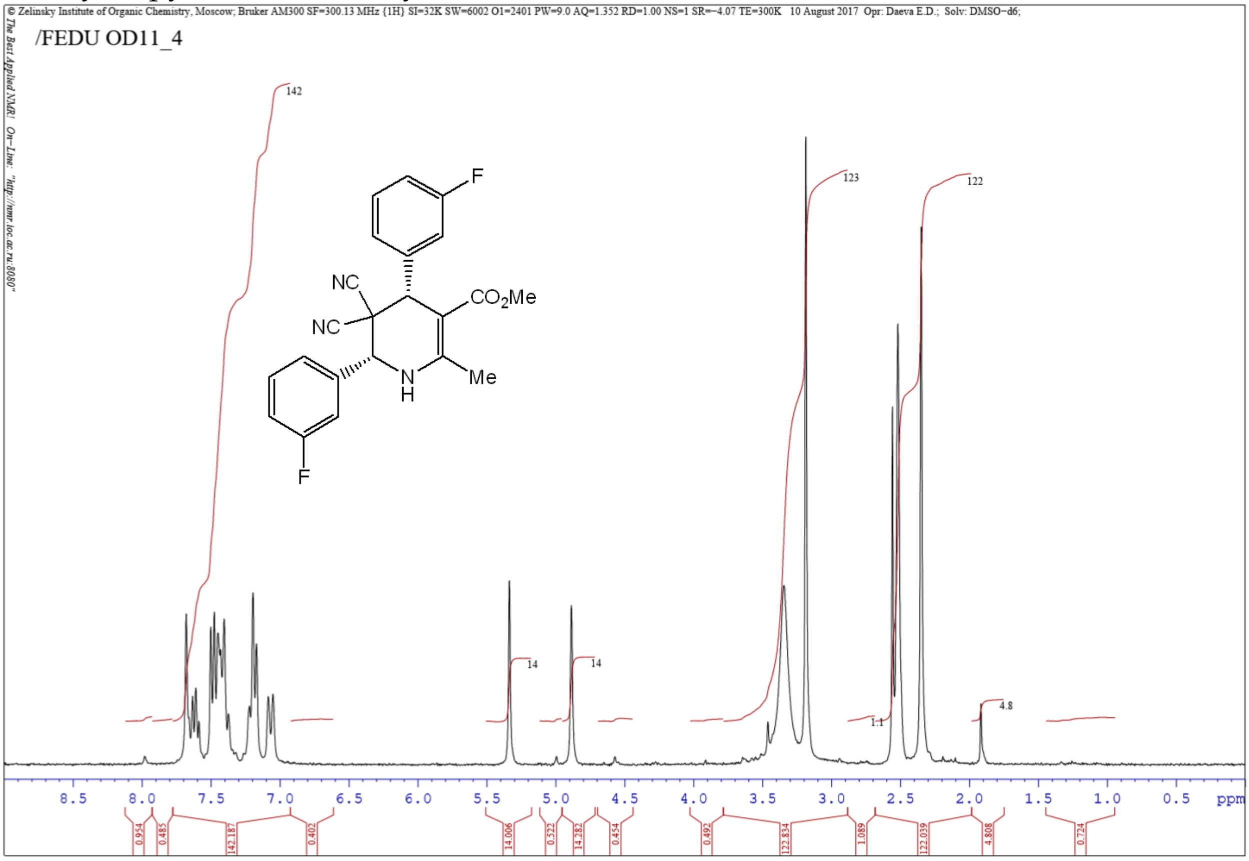

$^{1}$H NMR of methyl (4*SR*, 6*RS*)-5,5-dicyano-2-methyl-4,6-bis(3-chloro)phenyl-1,4,5,6-tetrahydropyridine-3-carboxylate (**4f**)

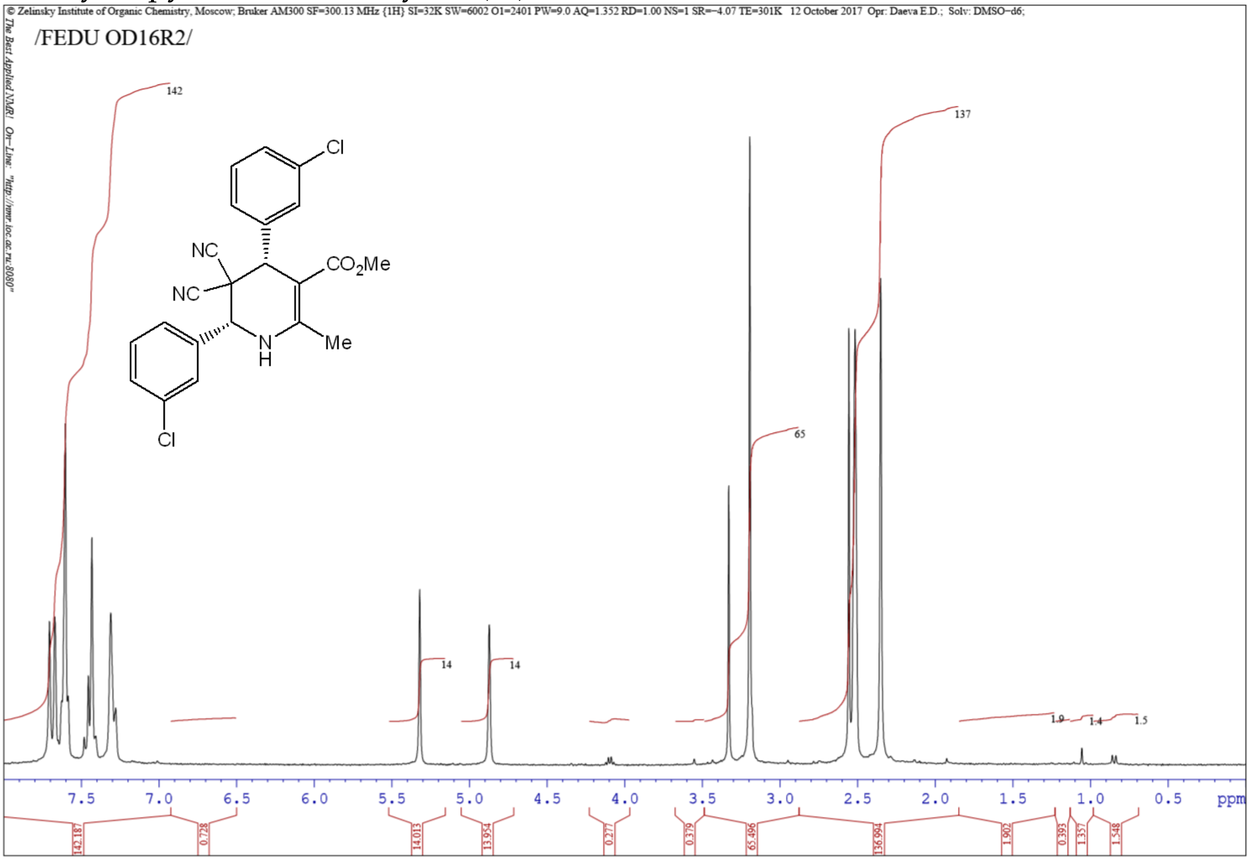


$^{13}$C NMR of **4f**

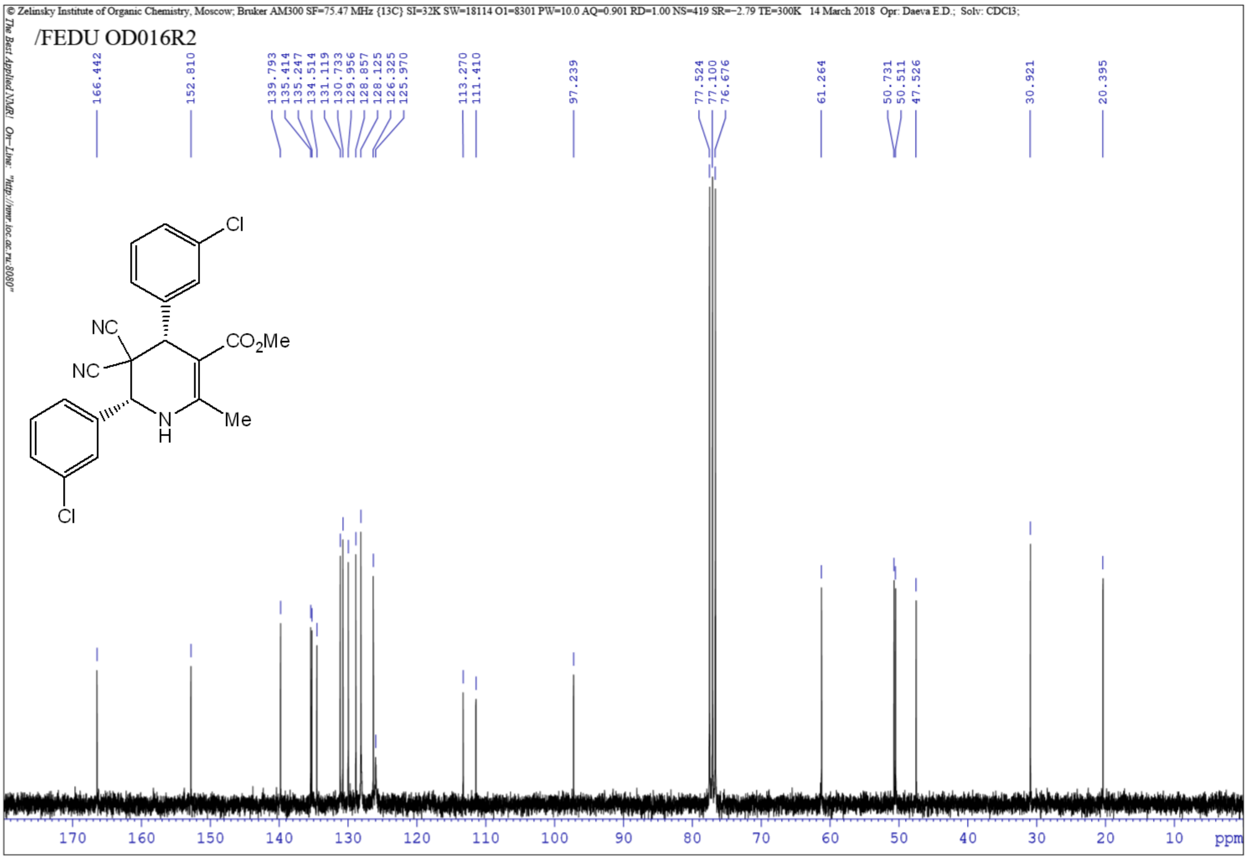

$^1$H NMR of methyl (4*SR*, 6*RS*)-5,5-dicyano-2-methyl-4,6-(3-pyridine)-1,4,5,6-tetrahydropyridine-3-carboxylate (**4g**)

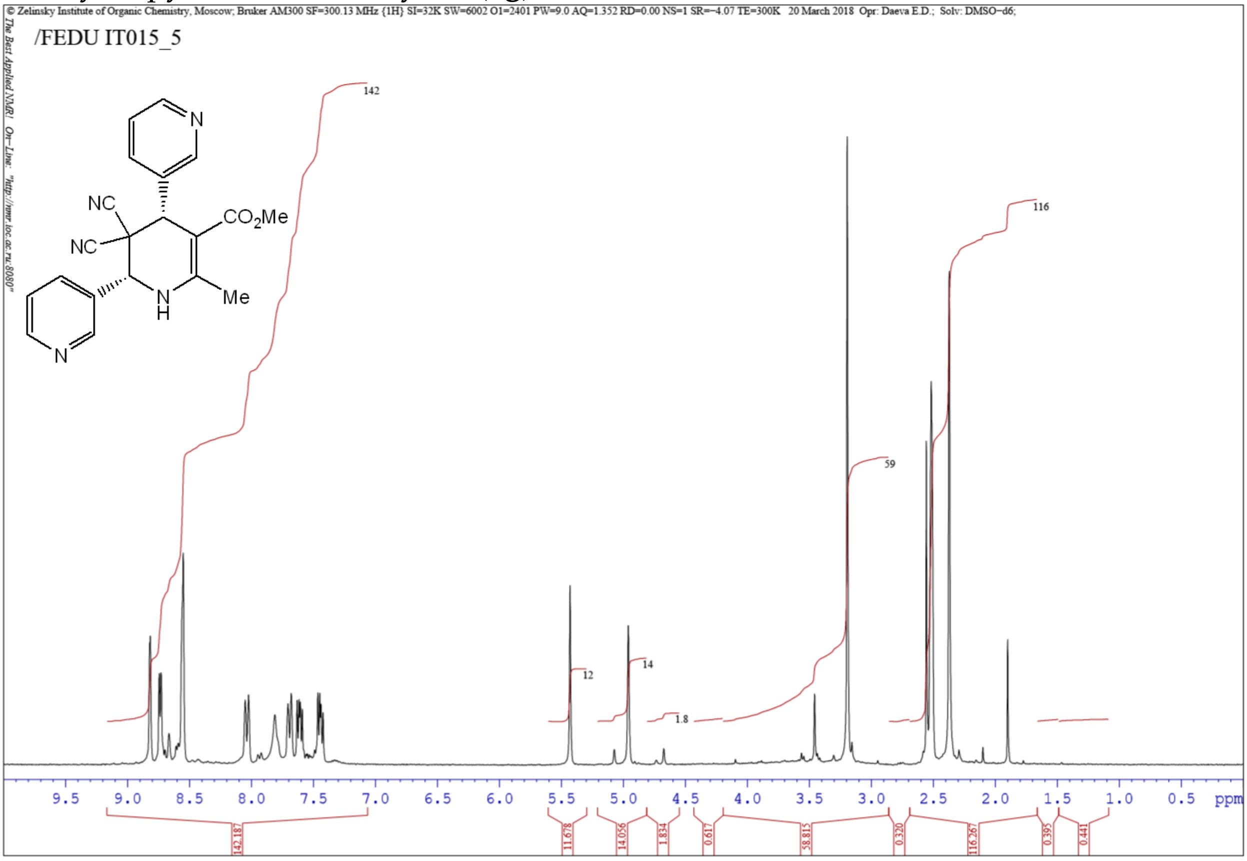


$^{13}$C NMR of **4g**

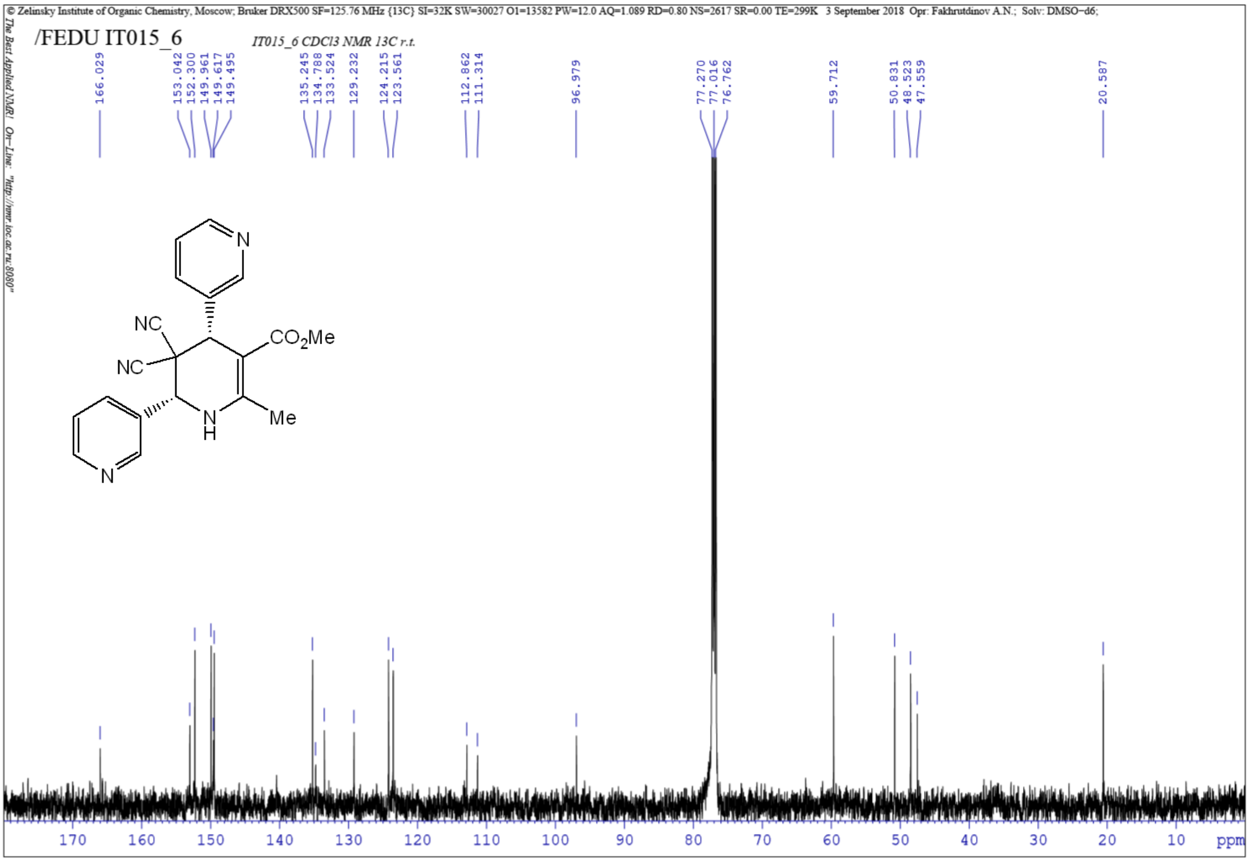

$^{1}H$ NMR of ethyl (4*SR*, 6*RS*)-5,5-dicyano-2-methyl-4,6-diphenyl-1,4,5,6-tetrahydropyridine-3-carboxylate (**4h**)

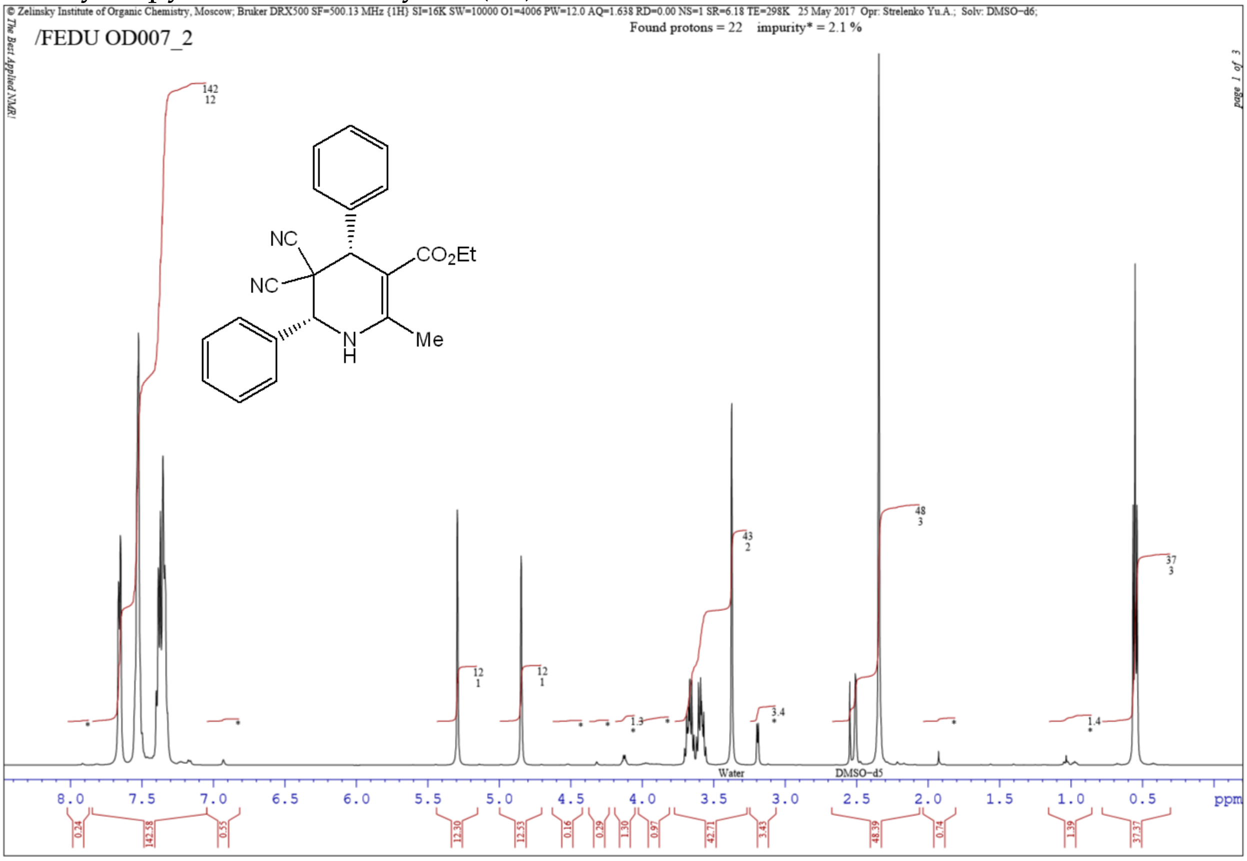


$^{13}C$ NMR of **4h**

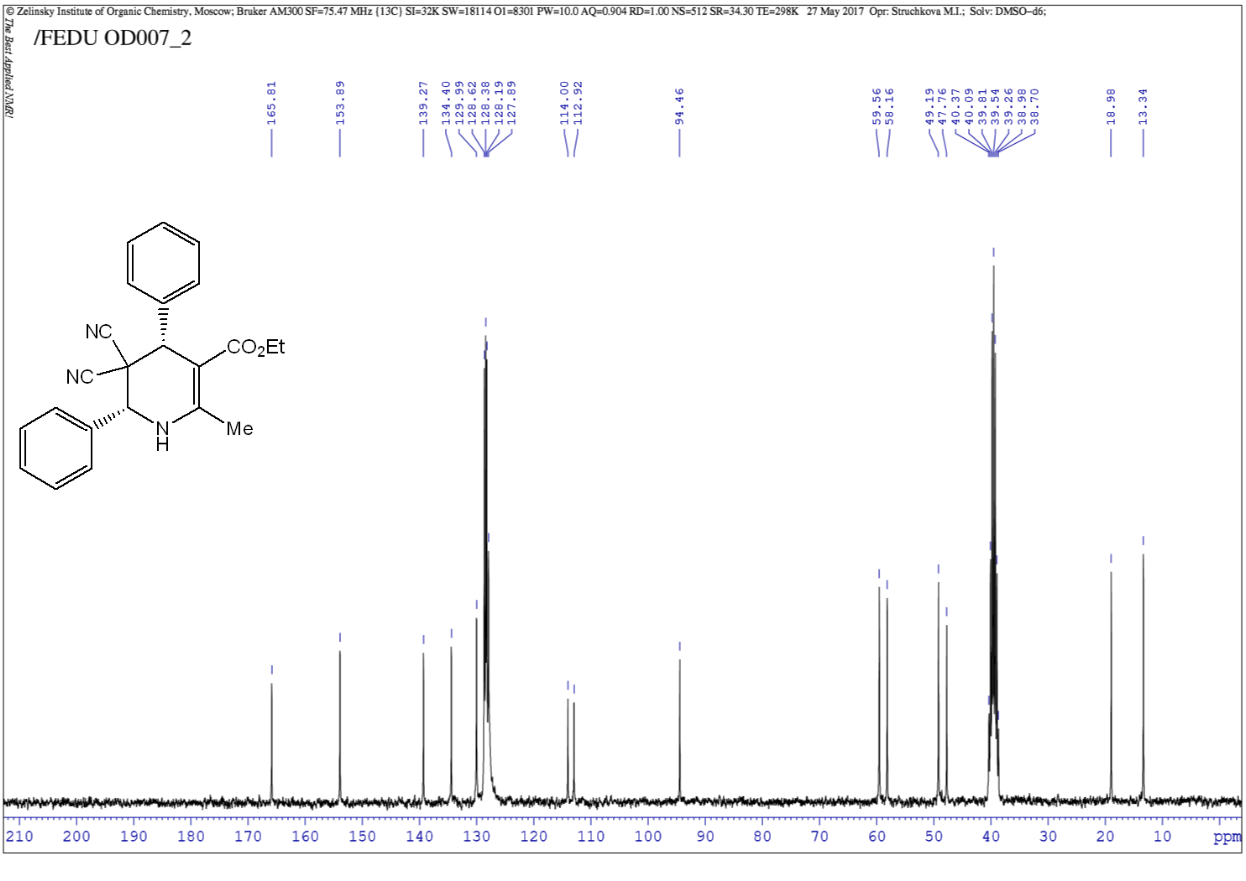

$^{1}$H NMR of ethyl (4*SR*, 6*RS*)-5,5-dicyano-2-methyl-4,6-bis(4-fluoro)phenyl-1,4,5,6-tetrahydropyridine-3-carboxylate (**4i**)

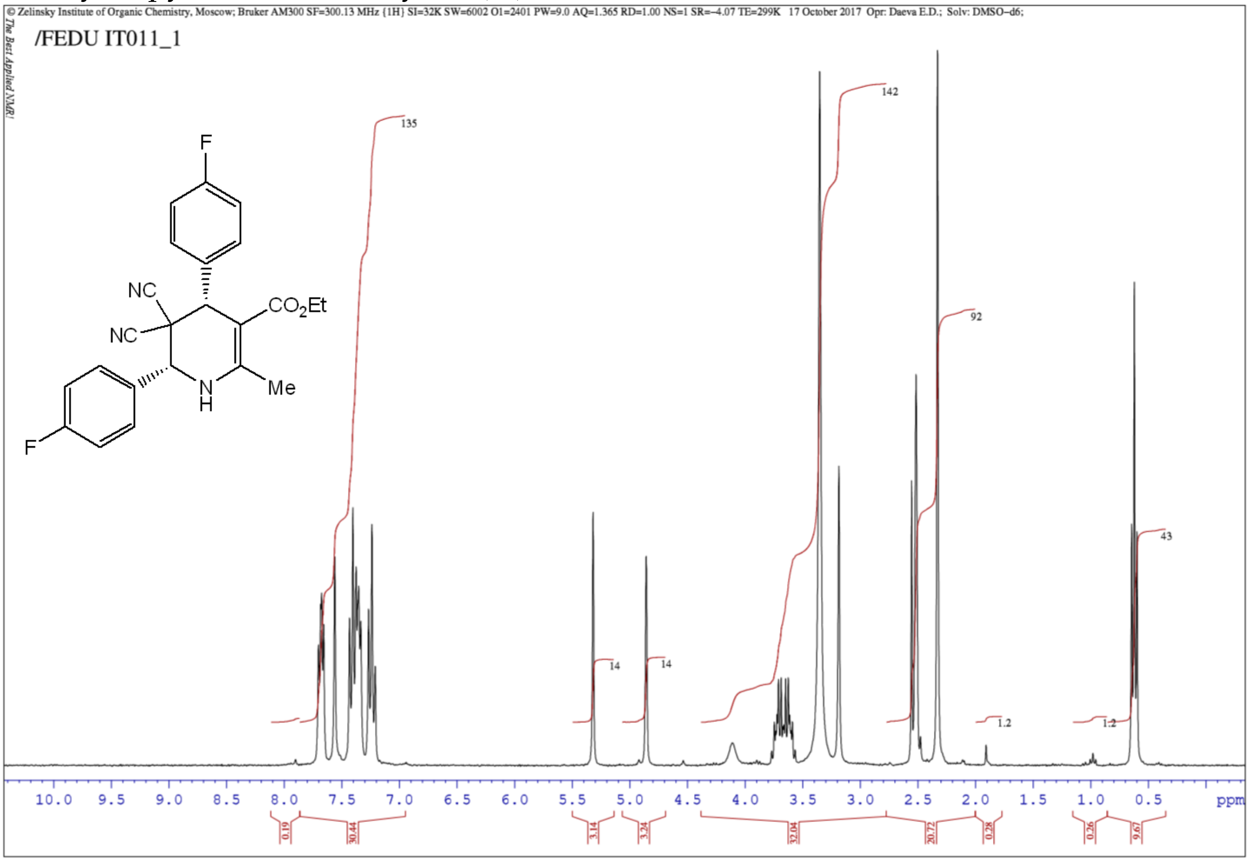


$^{13}$C NMR of **4i**

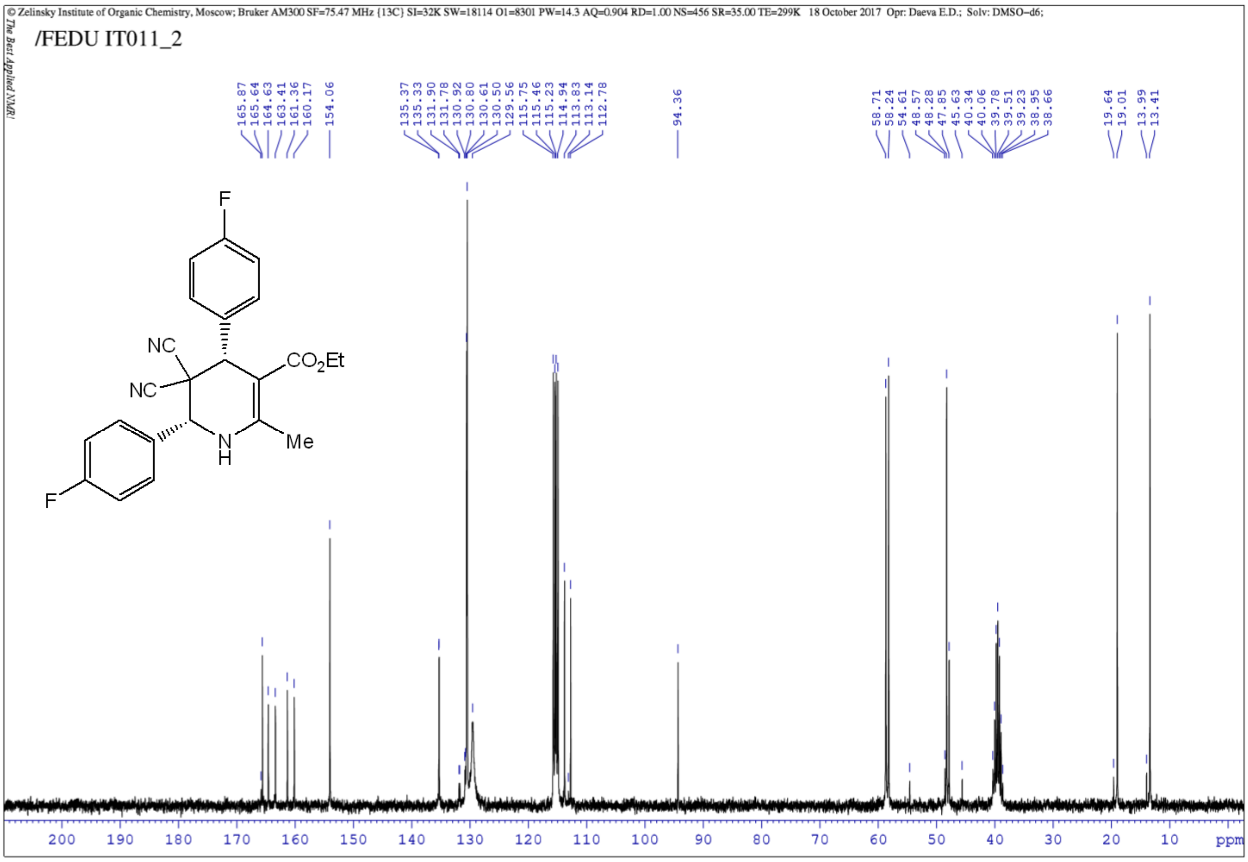

$^{1}$H NMR of ethyl (4*SR*, 6*RS*)-5,5-dicyano-2-methyl-4,6-bis(4-nitro)phenyl-1,4,5,6-tetrahydropyridine-3-carboxylate (**4j**)

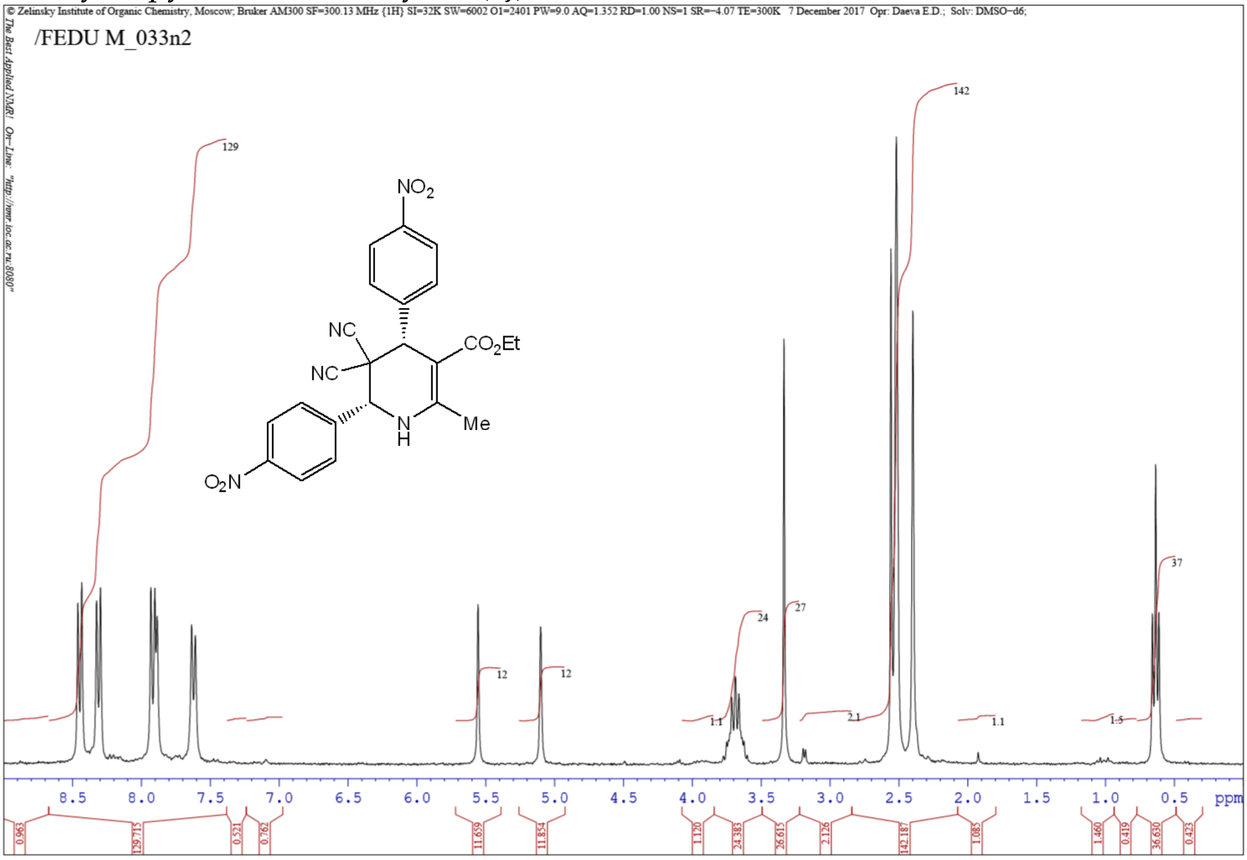


$^{13}$C NMR of **4j**

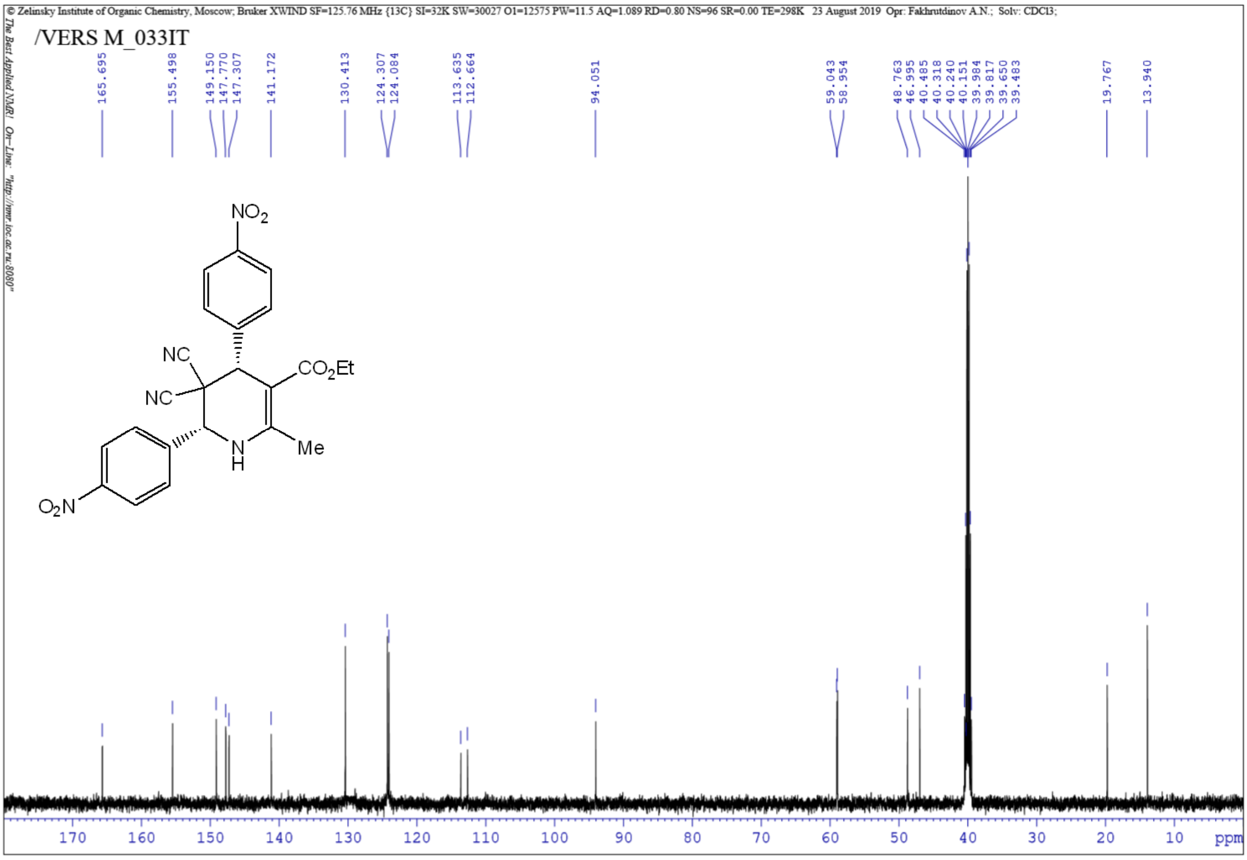

$^{1}$H NMR of methyl (4*SR*, 6*RS*)-5,5-dicyano-2-ethyl-4,6-diphenyl-1,4,5,6-tetrahydropyridine-3-carboxylate (**4k**)

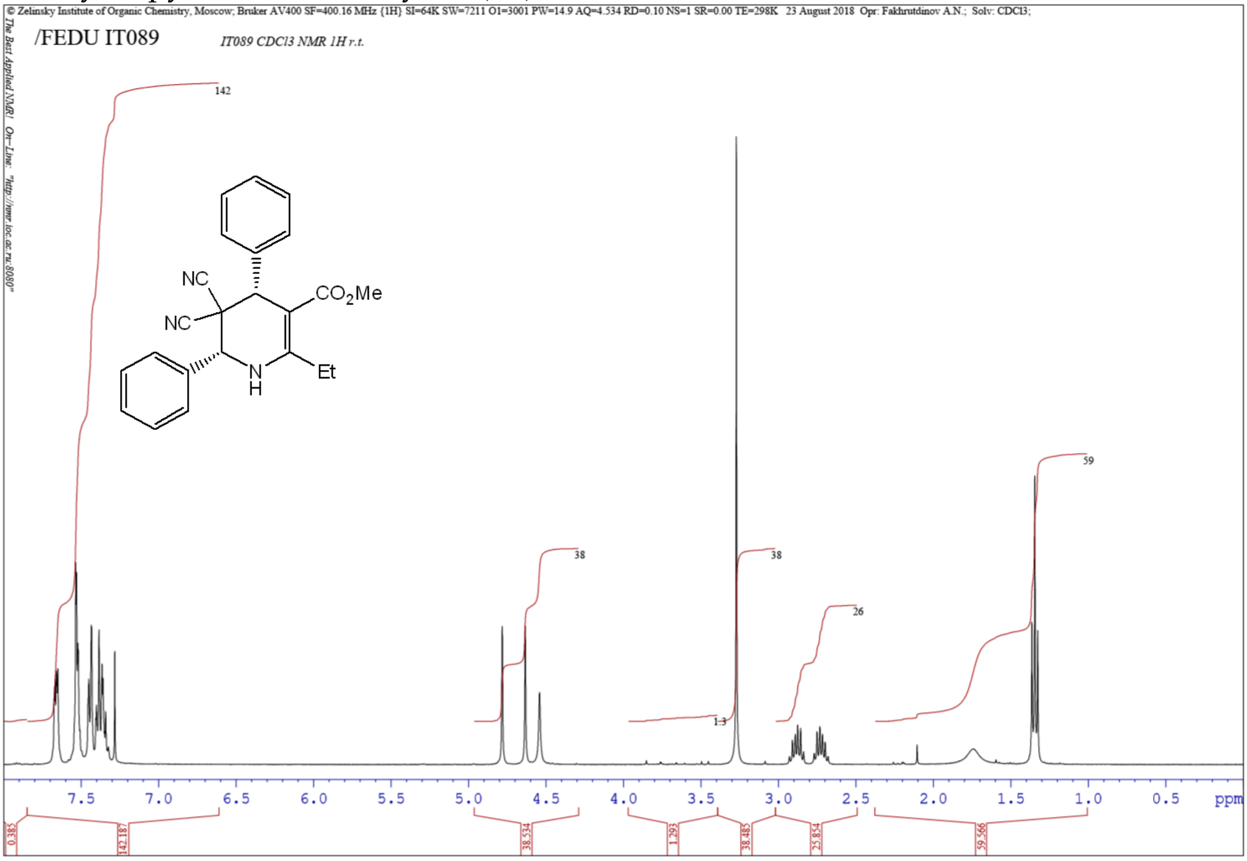

$^{1}$H NMR of methyl (4*SR*, 6*RS*)-5,5-dicyano-2-ethyl-4,6-bis(4-methyl)phenyl-1,4,5,6-tetrahydropyridine-3-carboxylate (**4l**)

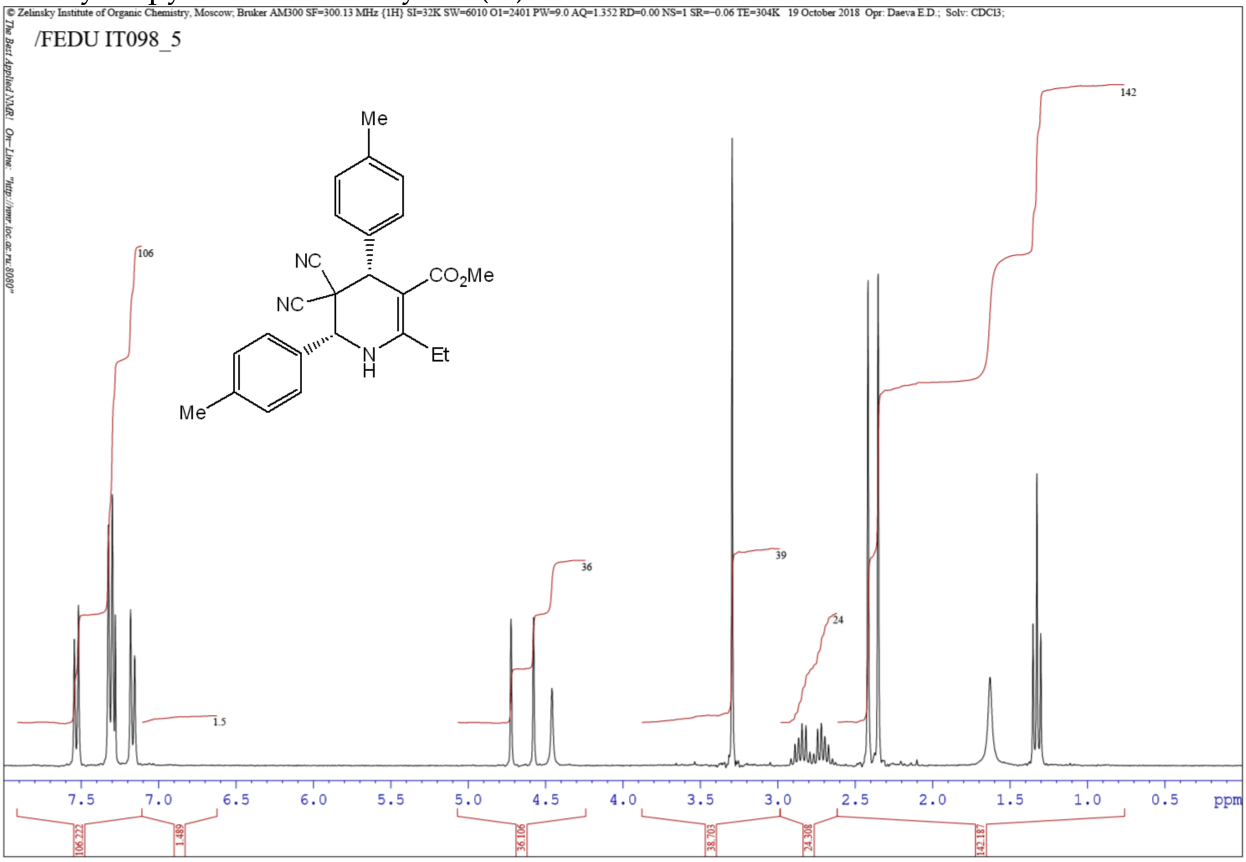


$^{13}$C NMR of **4l**

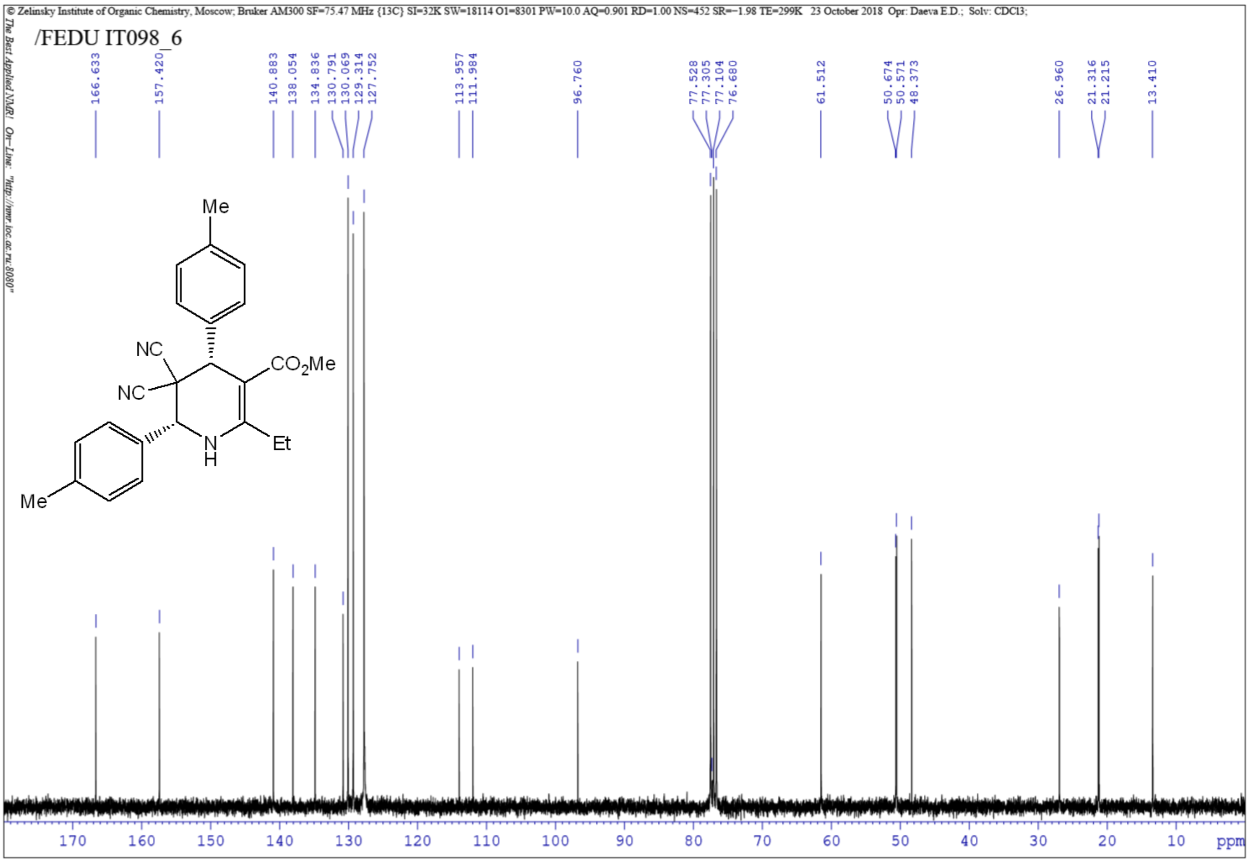

$^{1}$H NMR of methyl (4*SR*, 6*RS*)-5,5-dicyano-2-ethyl-4,6-bis(4-bromo)phenyl-1,4,5,6-tetrahydropyridine-3-carboxylate (**4m**)

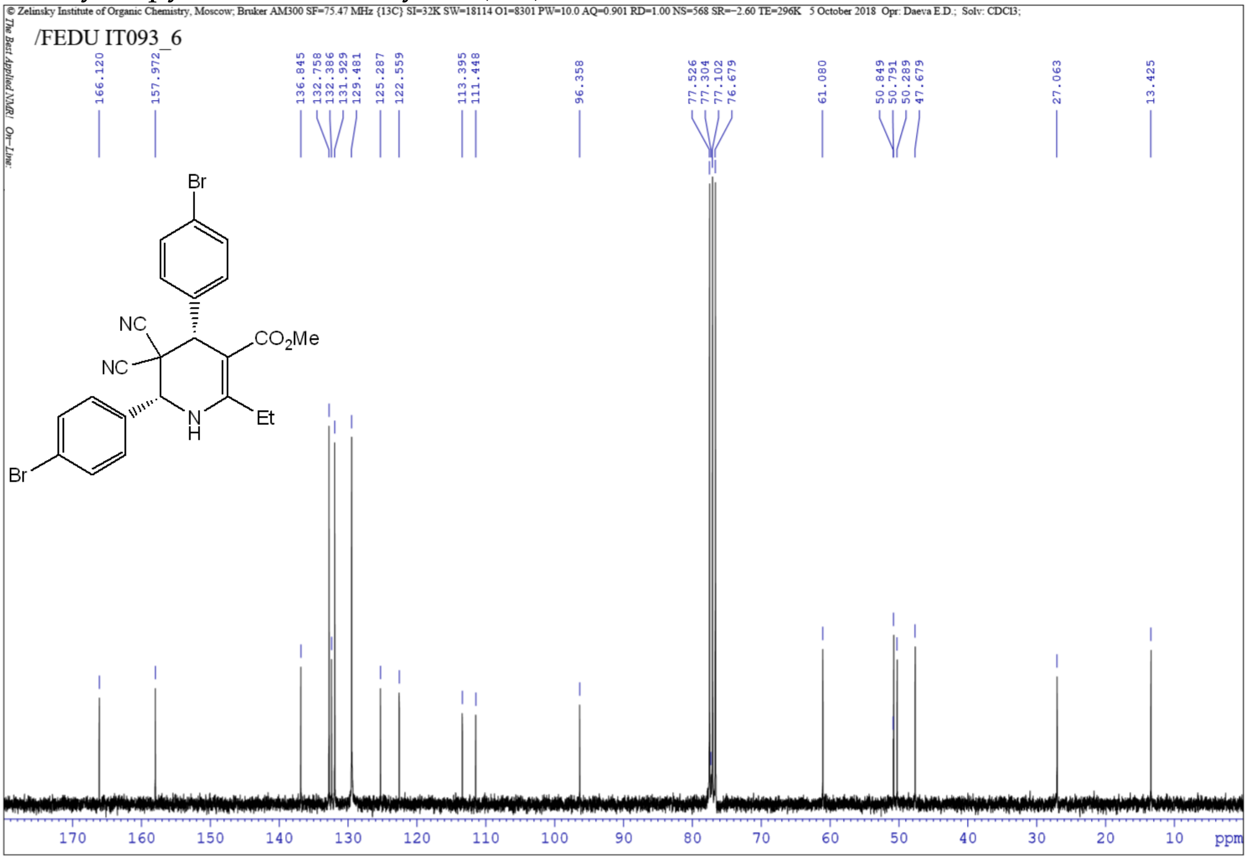

$^1$H NMR of methyl (4*SR*, 6*RS*)-5,5-dicyano-2-ethyl-4,6-bis(4-nitro)phenyl-1,4,5,6-tetrahydropyridine-3-carboxylate (**4n**)

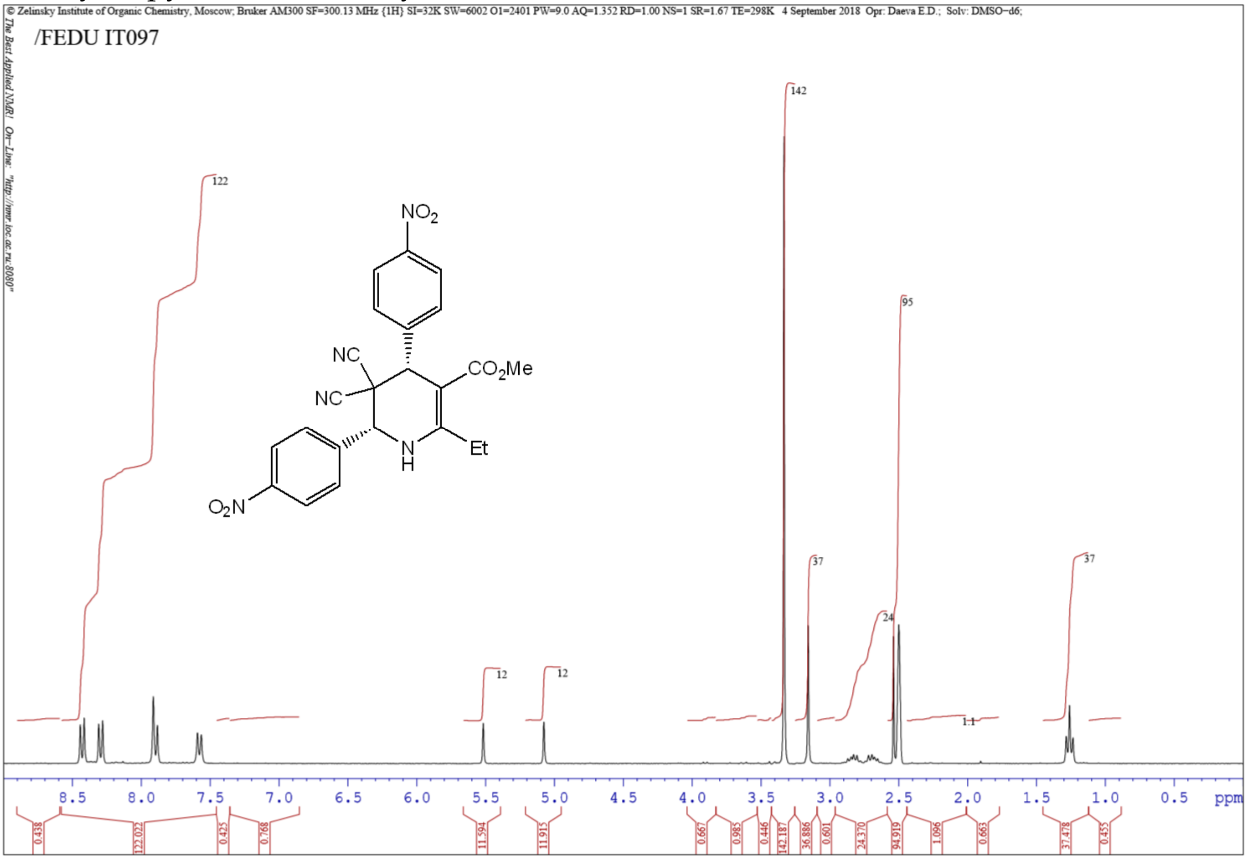


$^{13}$C NMR of **4n**

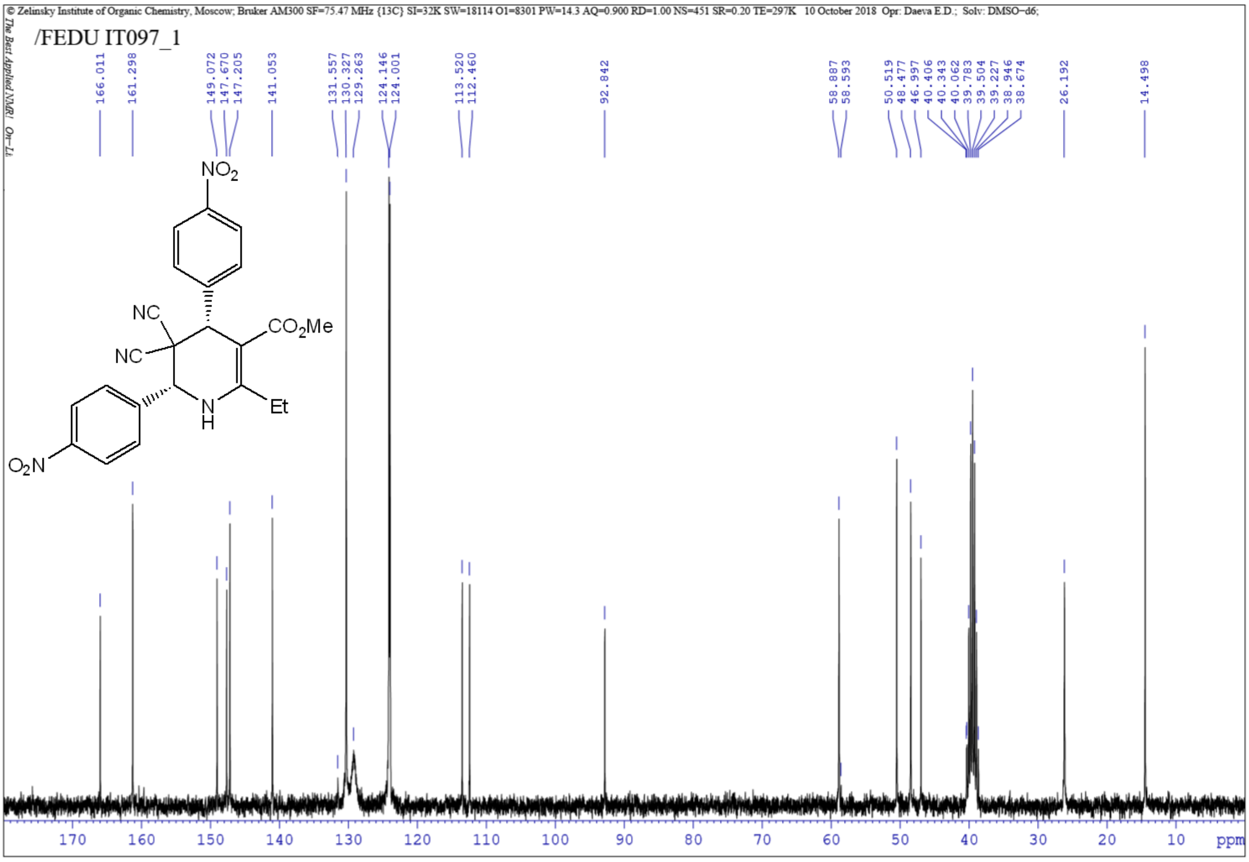

$^{1}$H NMR of methyl (4*SR*, 6*RS*)-5,5-dicyano-2-phenyl-4,6-diphenyl-1,4,5,6-tetrahydropyridine-3-carboxylate (**4o**)

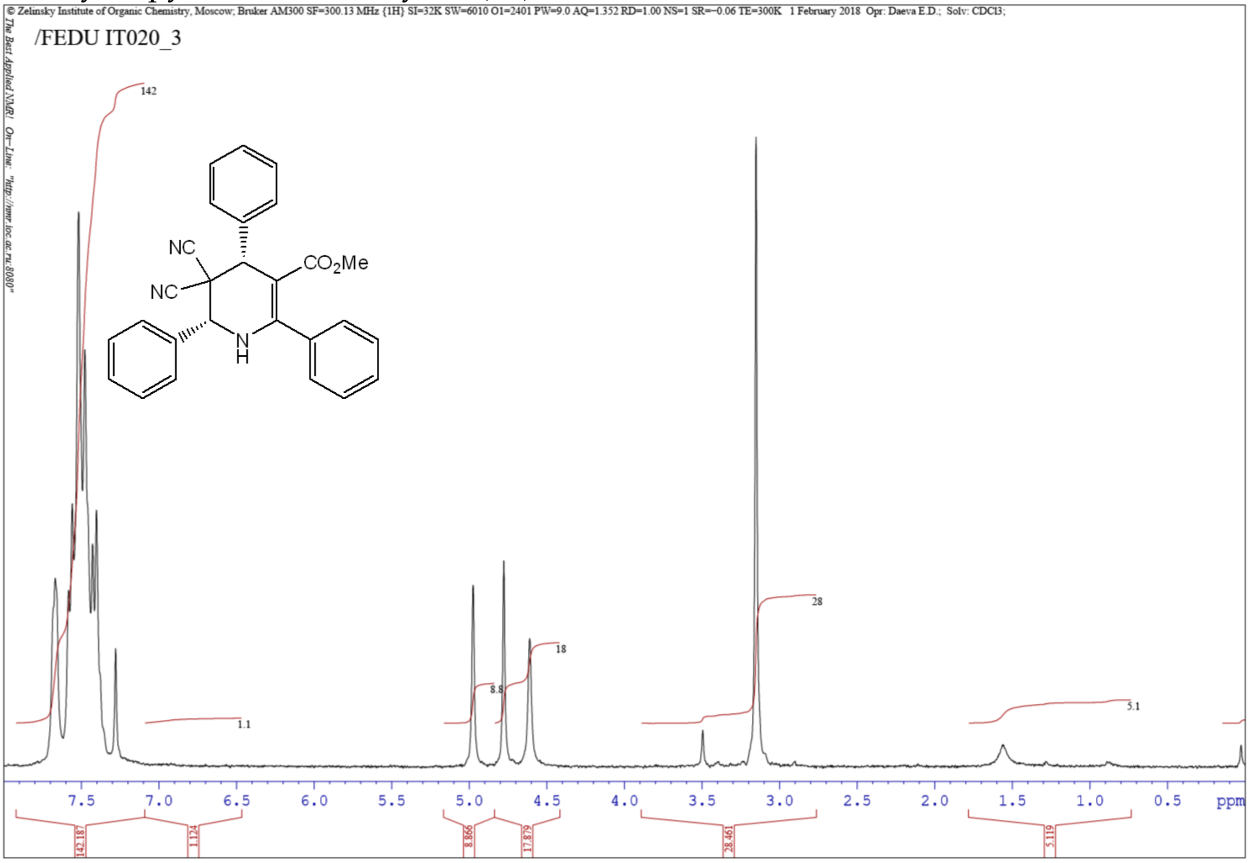

$^{1}$H NMR of methyl (4*SR*, 6*RS*)-5,5-dicyano-2-phenyl-4,6-bis(3-fluoro)phenyl-1,4,5,6-tetrahydropyridine-3-carboxylate (**4p**)

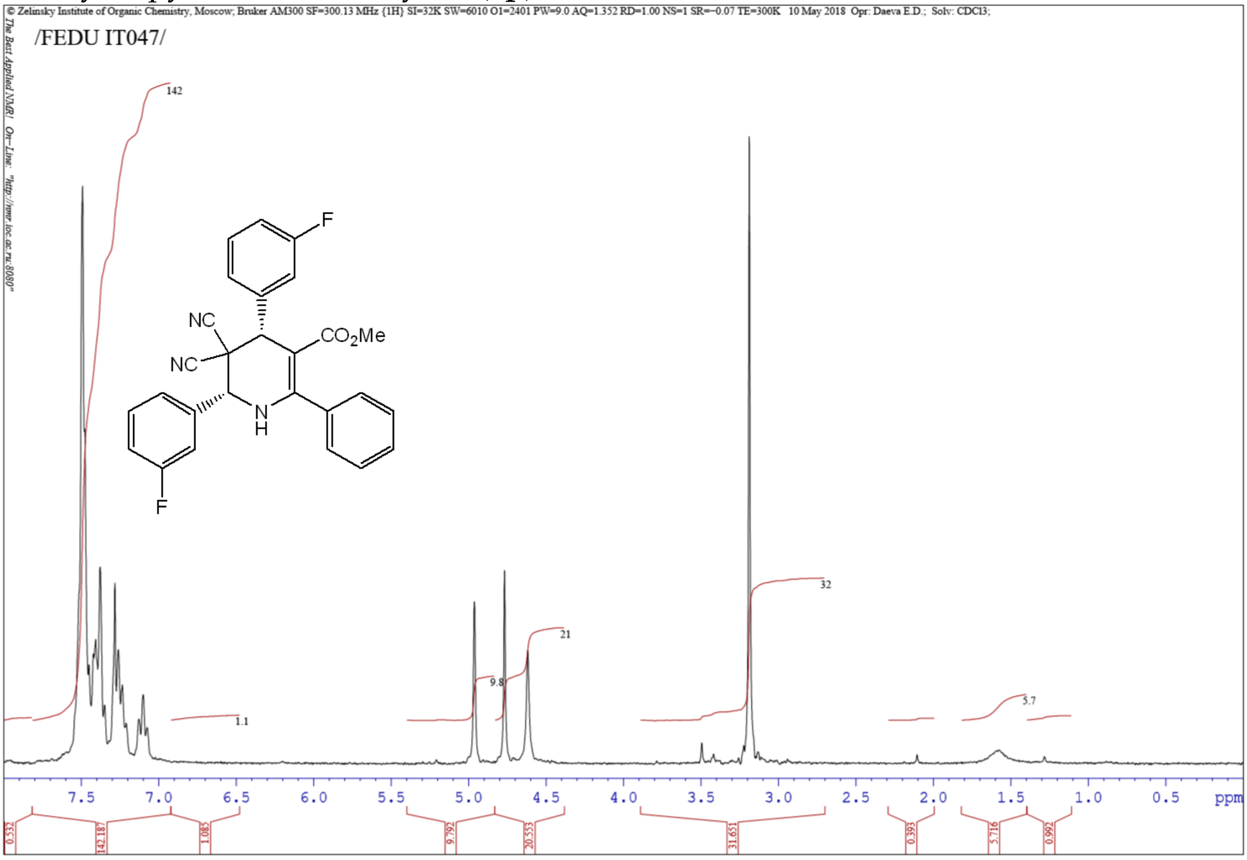


$^{13}$C NMR of **4p**

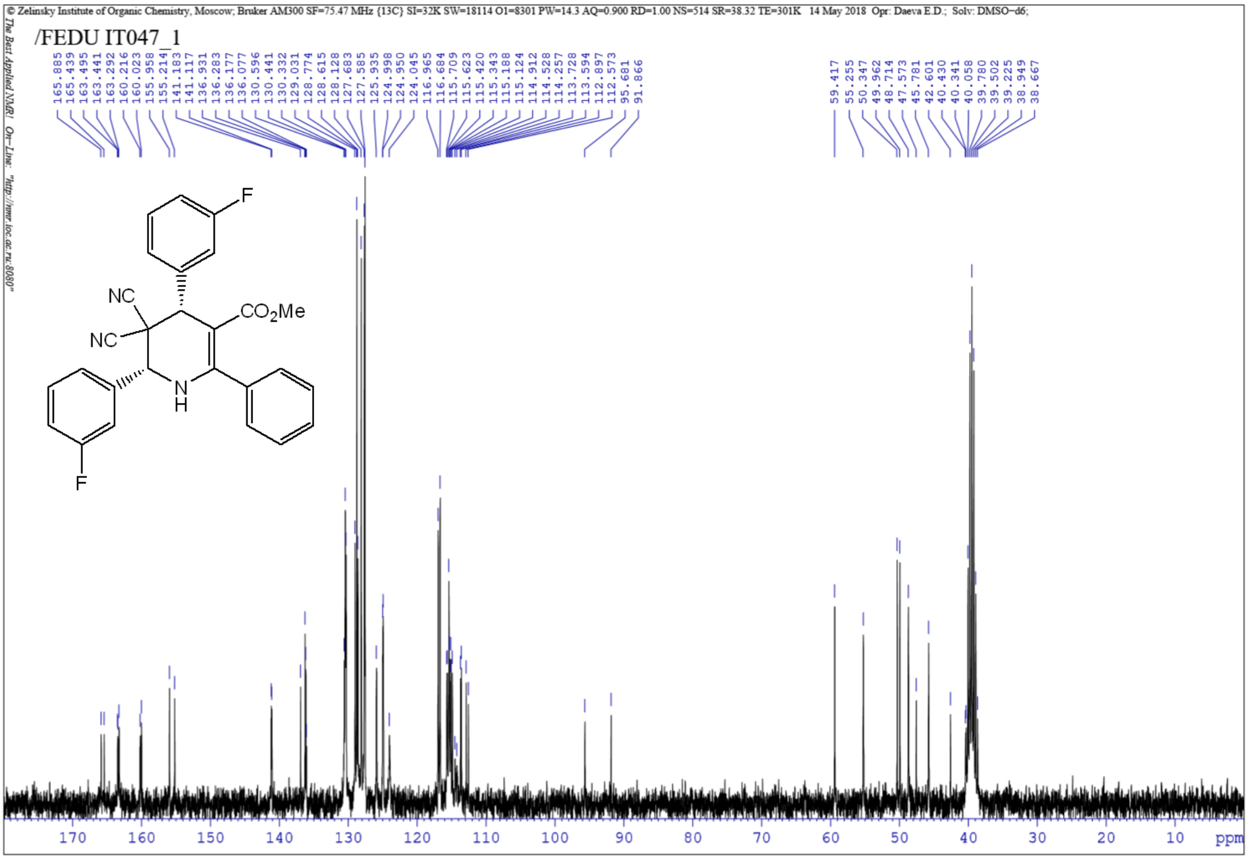

$^{1}$H NMR of ethyl (4*SR*, 6*RS*)-5,5-dicyano-2-phenyl-4,6-diphenyl-1,4,5,6-tetrahydropyridine-3-carboxylate (**4q**)

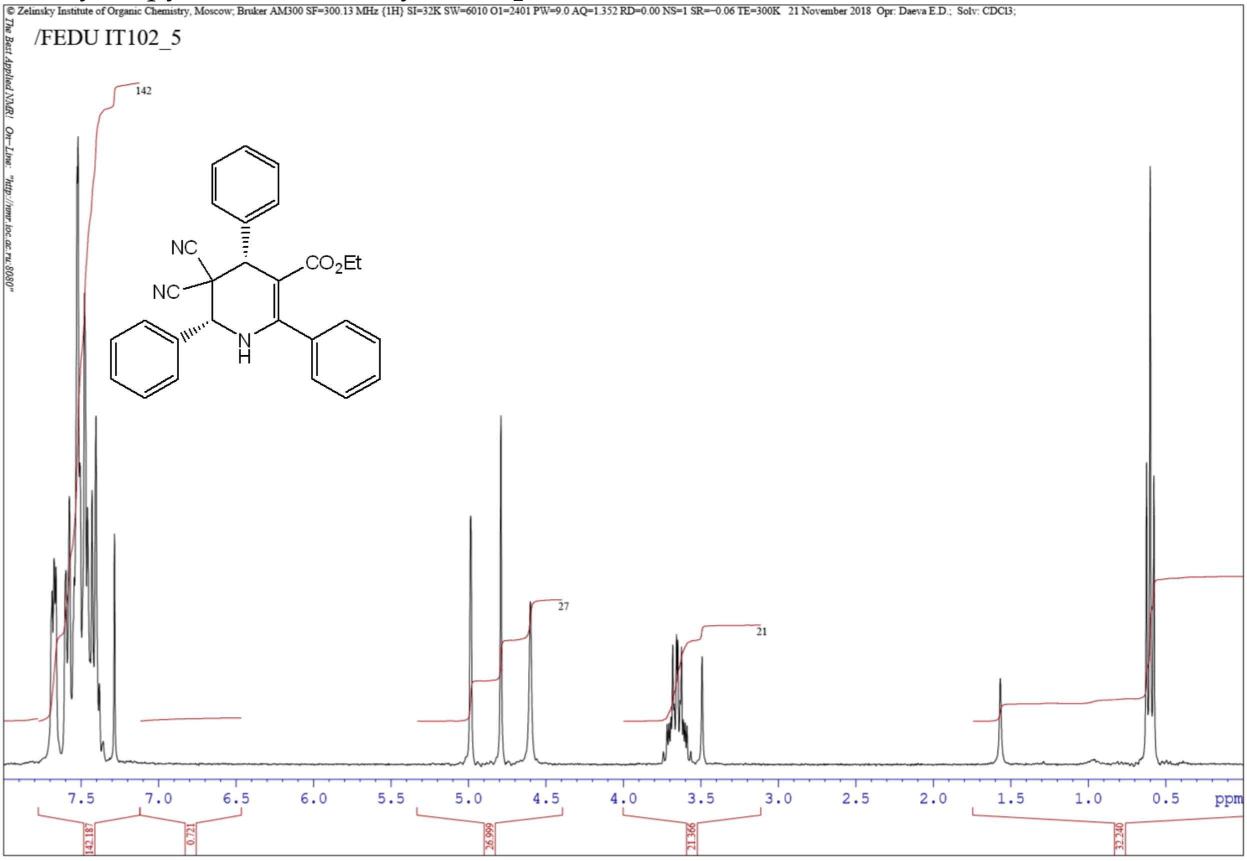


$^{13}$C NMR of **4q**

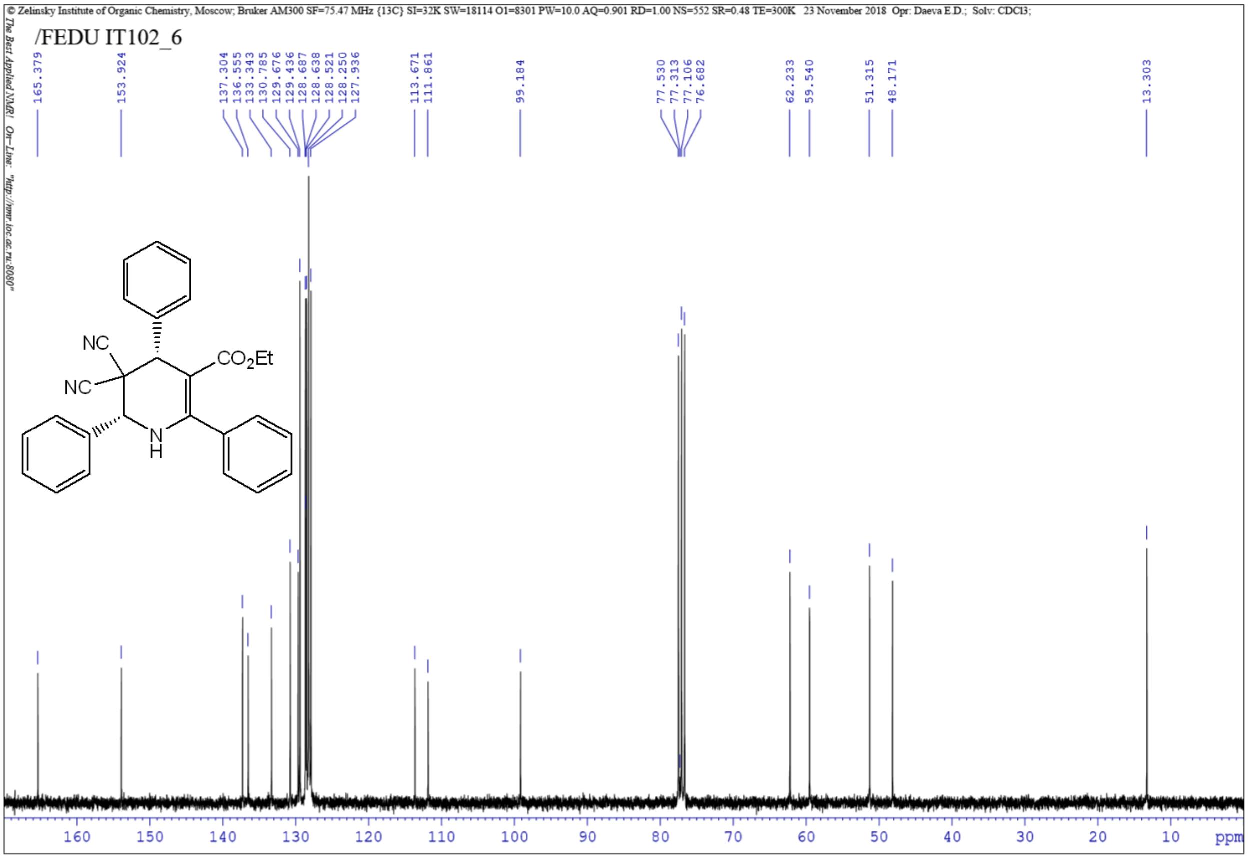

$^{1}$H NMR of ethyl (4*SR*, 6*RS*)-5,5-dicyano-2-phenyl-4,6-bis(4-methoxy)phenyl-1,4,5,6-tetrahydropyridine-3-carboxylate (**4r**)

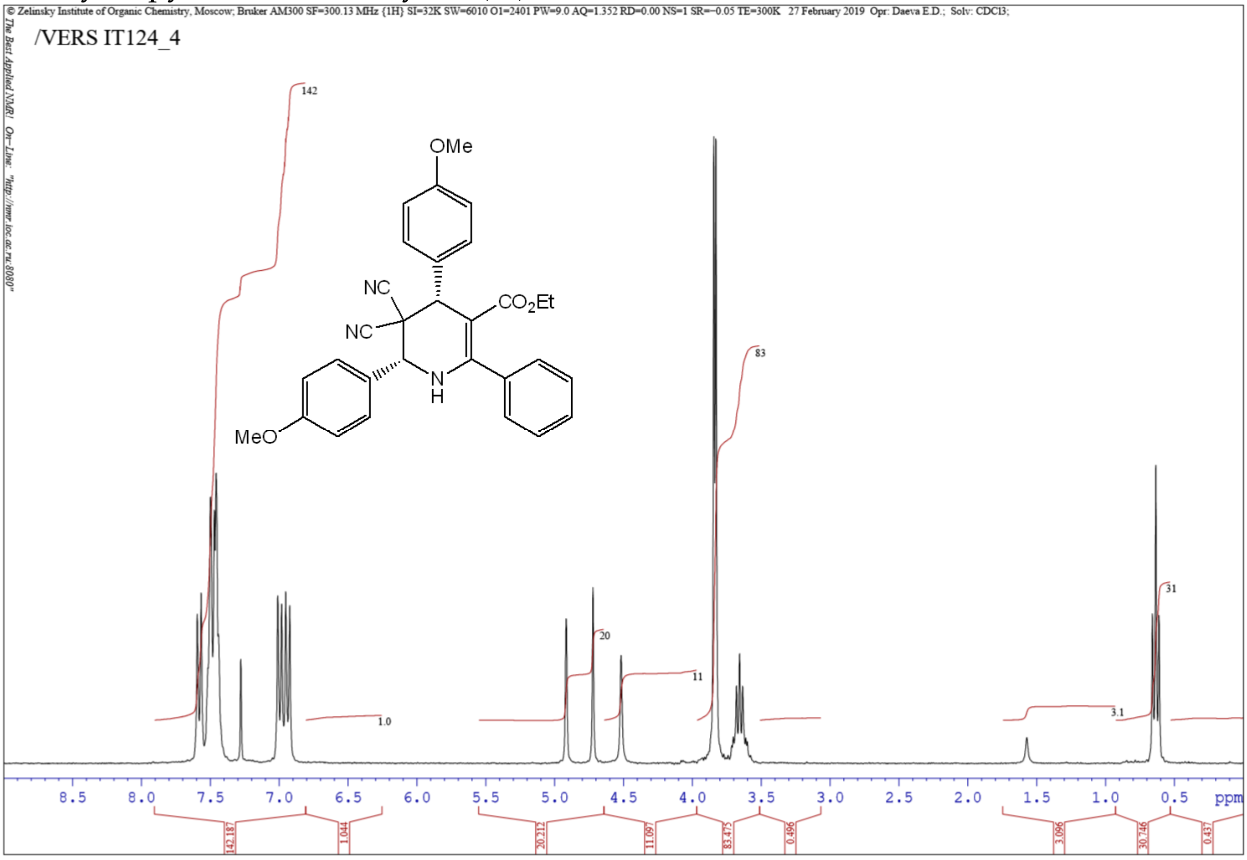

$^{13}$C NMR of **4r**

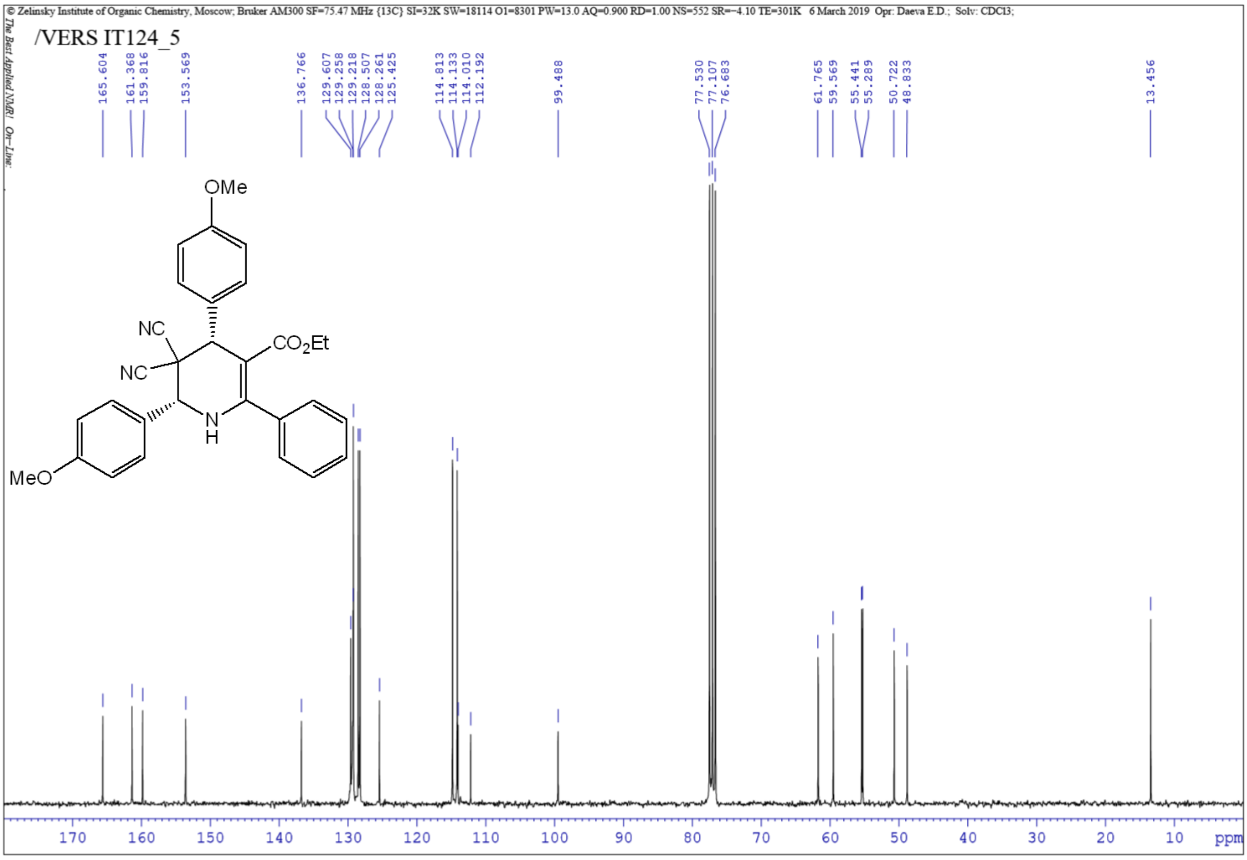

$^{1}$H NMR of methyl (4*SR*, 6*RS*)-5,5-dicyano-2-(4-bromo)phenyl-4,6-diphenyl-1,4,5,6-tetrahydropyridine-3-carboxylate (**4s**)

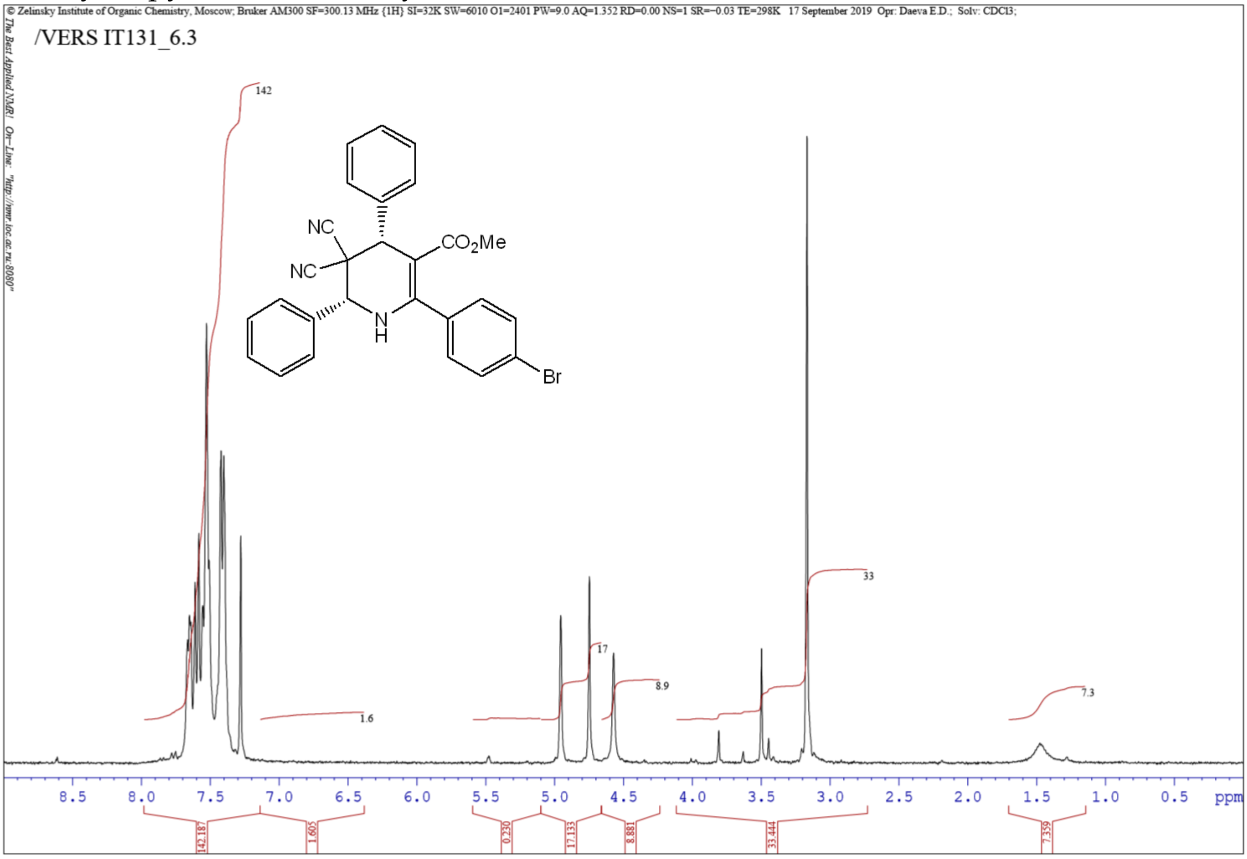

$^{1}$H NMR of methyl (4*SR*, 6*RS*)-5,5-dicyano-2-(4-bromo)phenyl-4,6-bis(4-methyl)phenyl-1,4,5,6-tetrahydropyridine-3-carboxylate (**4t**)

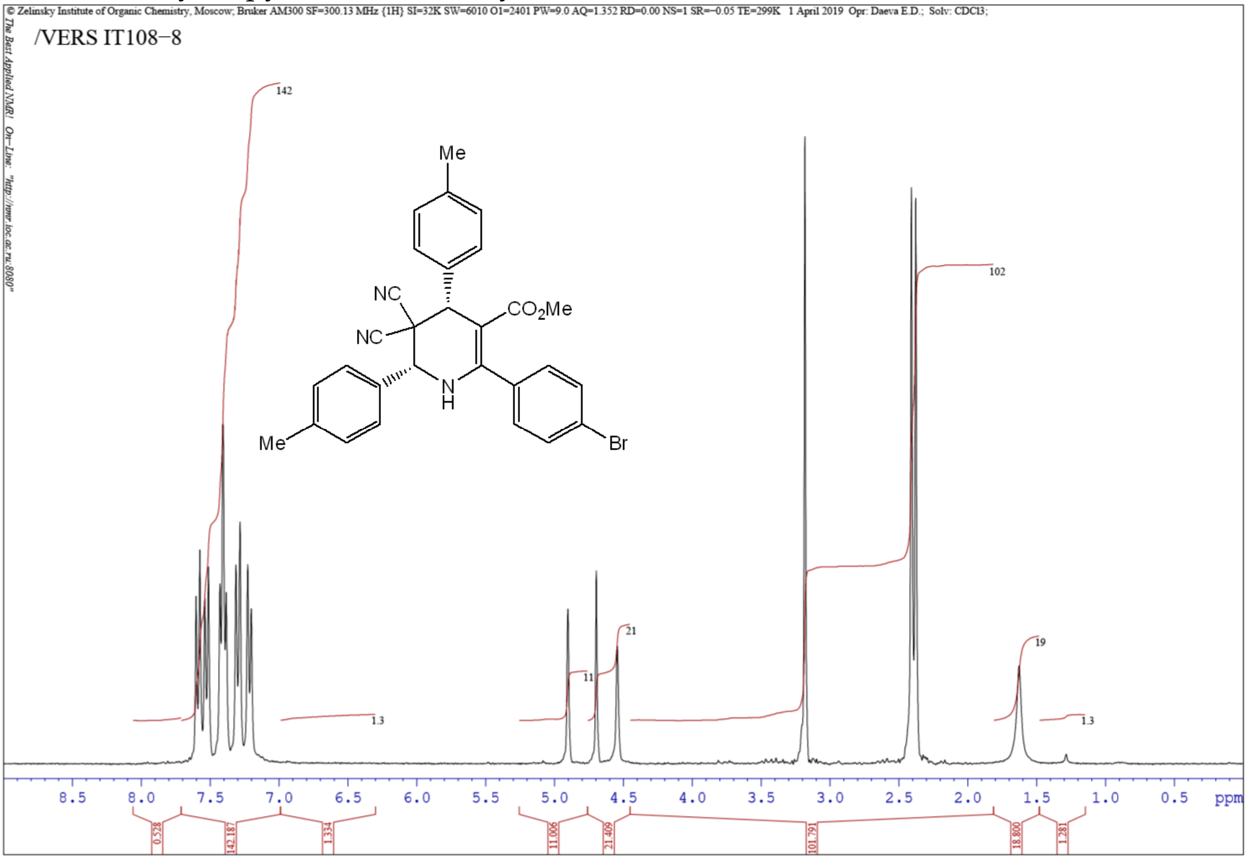


$^{13}$C NMR of **4t**

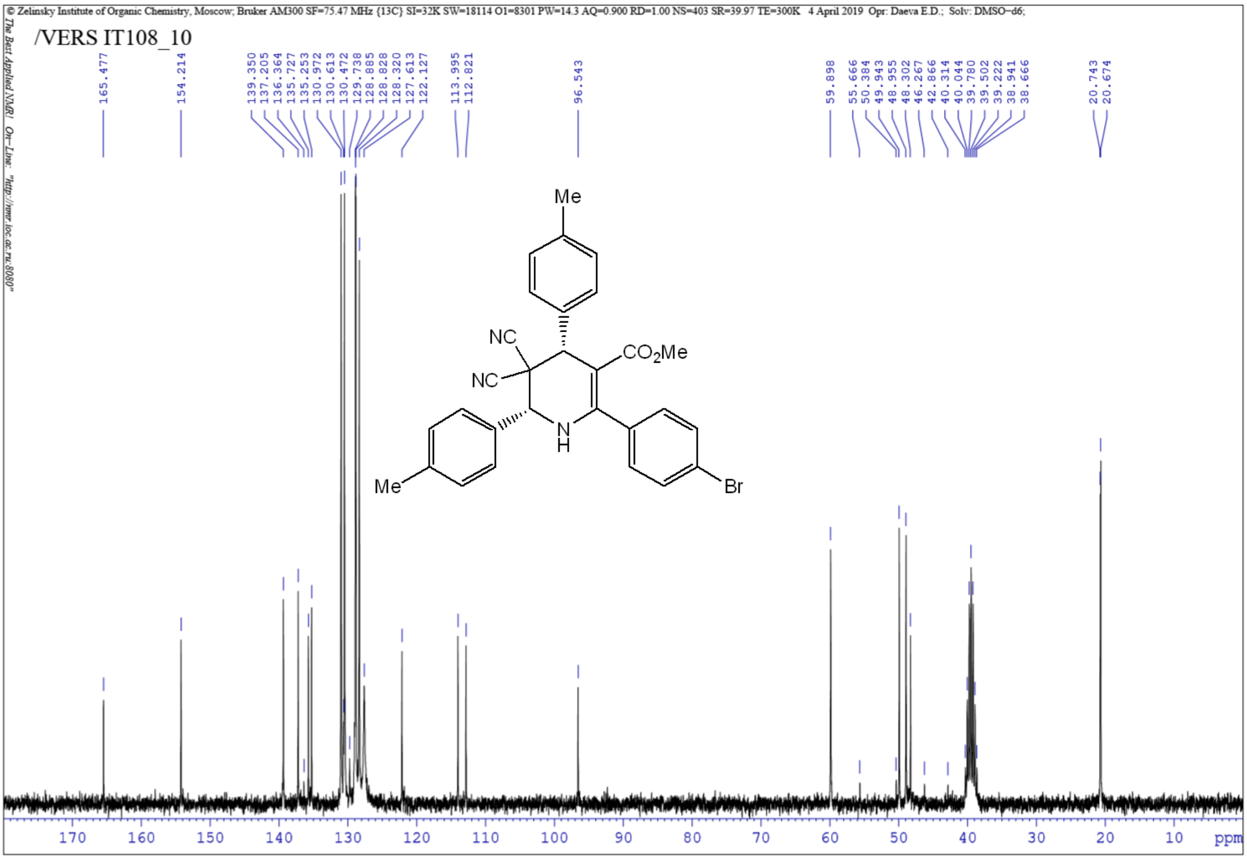

$^{1}$H NMR of 5-ethyl 3-methyl (4*RS*, 5*SR*, 6*RS*)-5-cyano-2-(4-bromo)phenyl-4,6-bis(4-bromo)phenyl-1,4,5,6-tetrahydropyridine-5,3-carboxylate (**5a**)

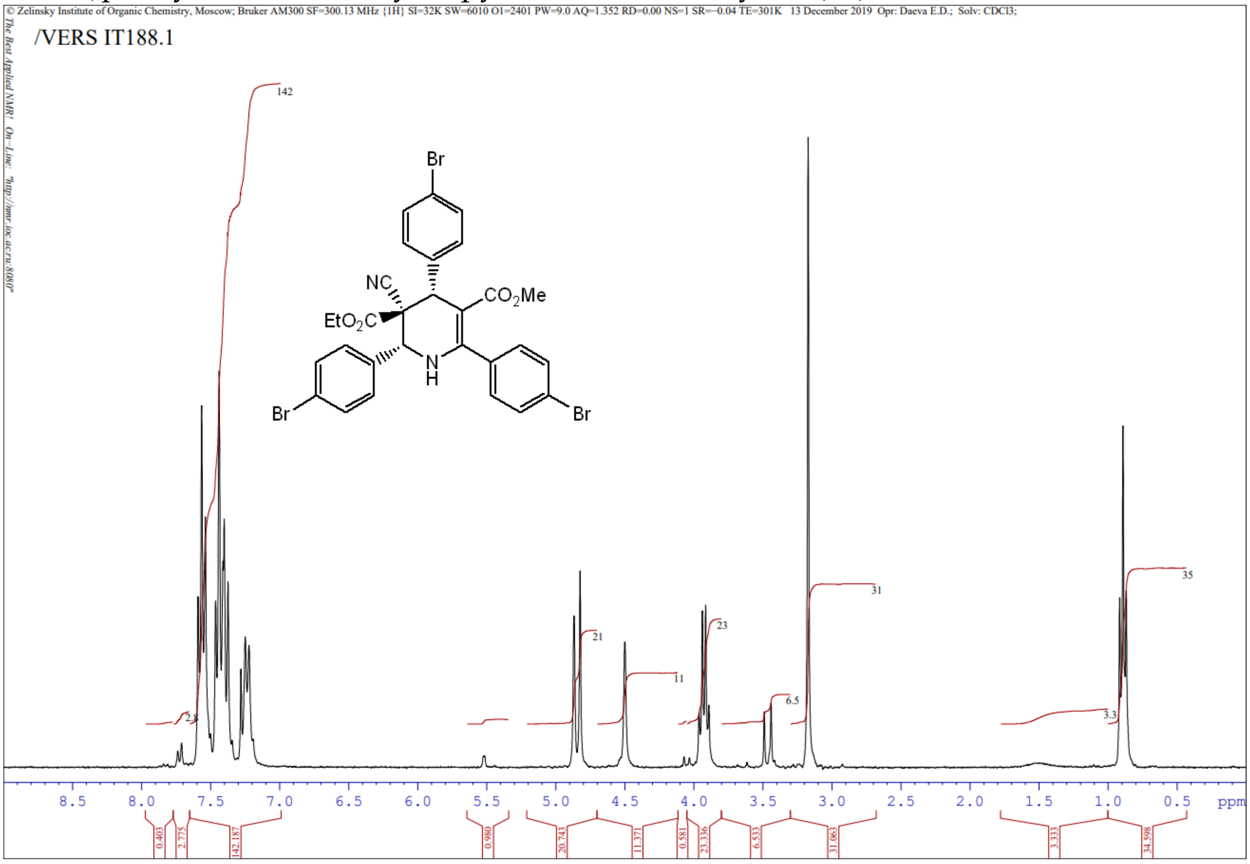


$^{13}$C NMR of **5a**

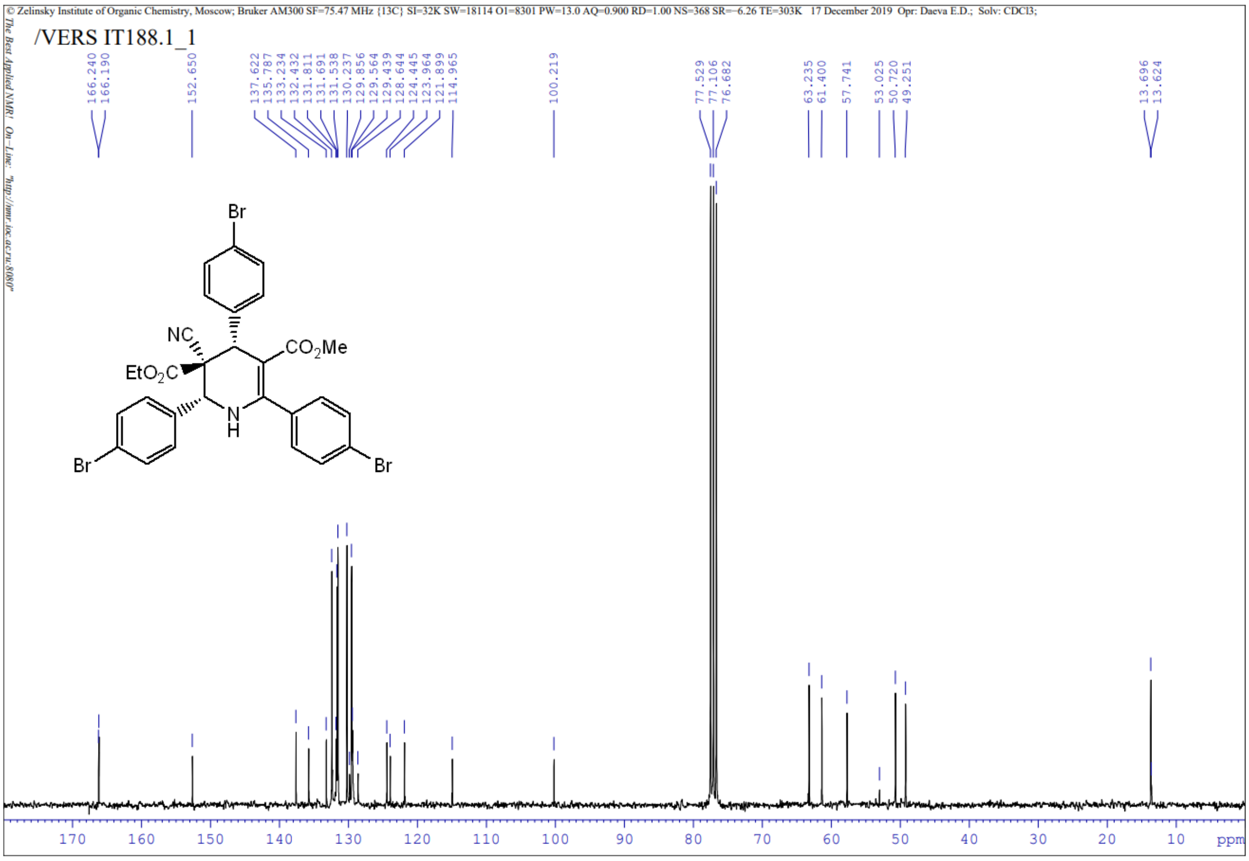

$^{1}$H NMR of 5-ethyl 3-methyl (4*RS*, 5*SR*, 6*RS*)-5-cyano-2-(4-chloro)phenyl-4,6-bis(4-bromo)phenyl-1,4,5,6-tetrahydropyridine-5,3-carboxylate (**5b**)

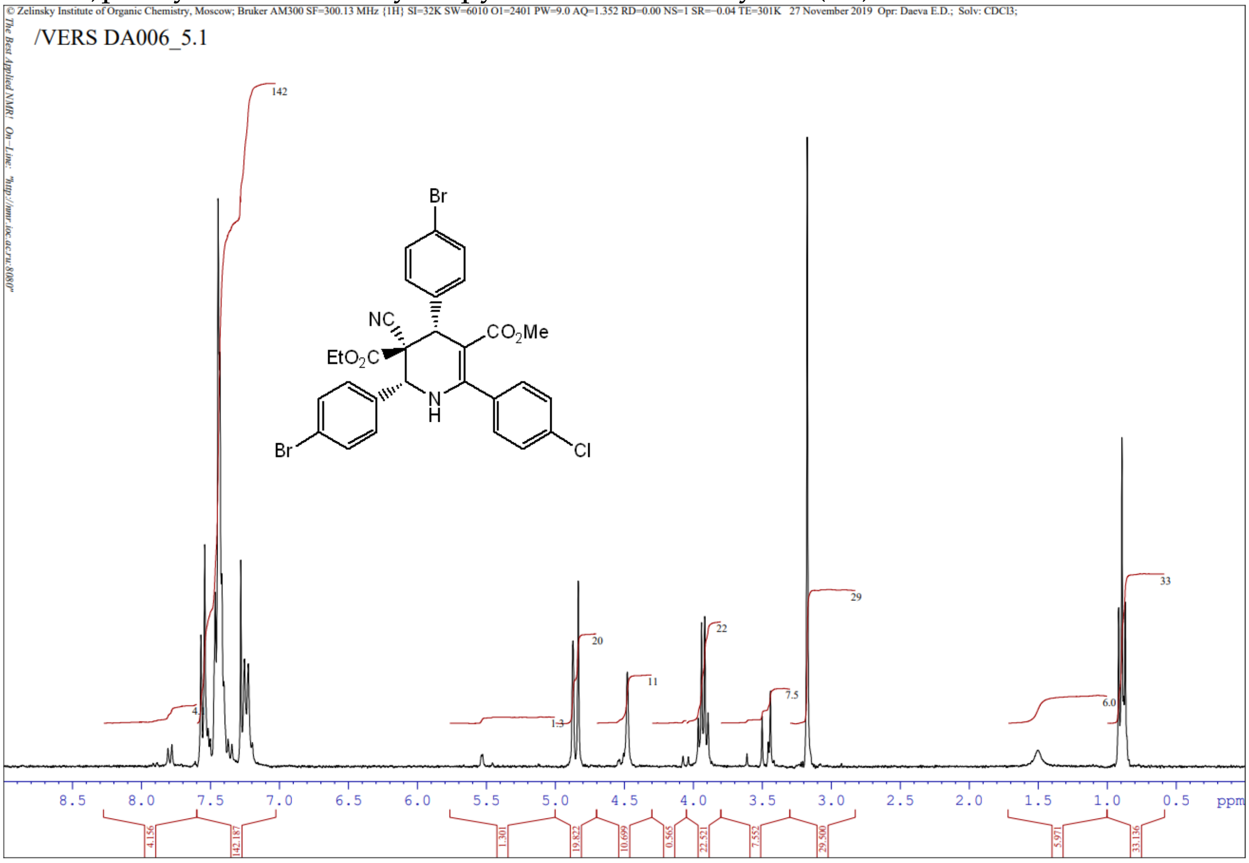


$^{13}$C NMR of **5b**

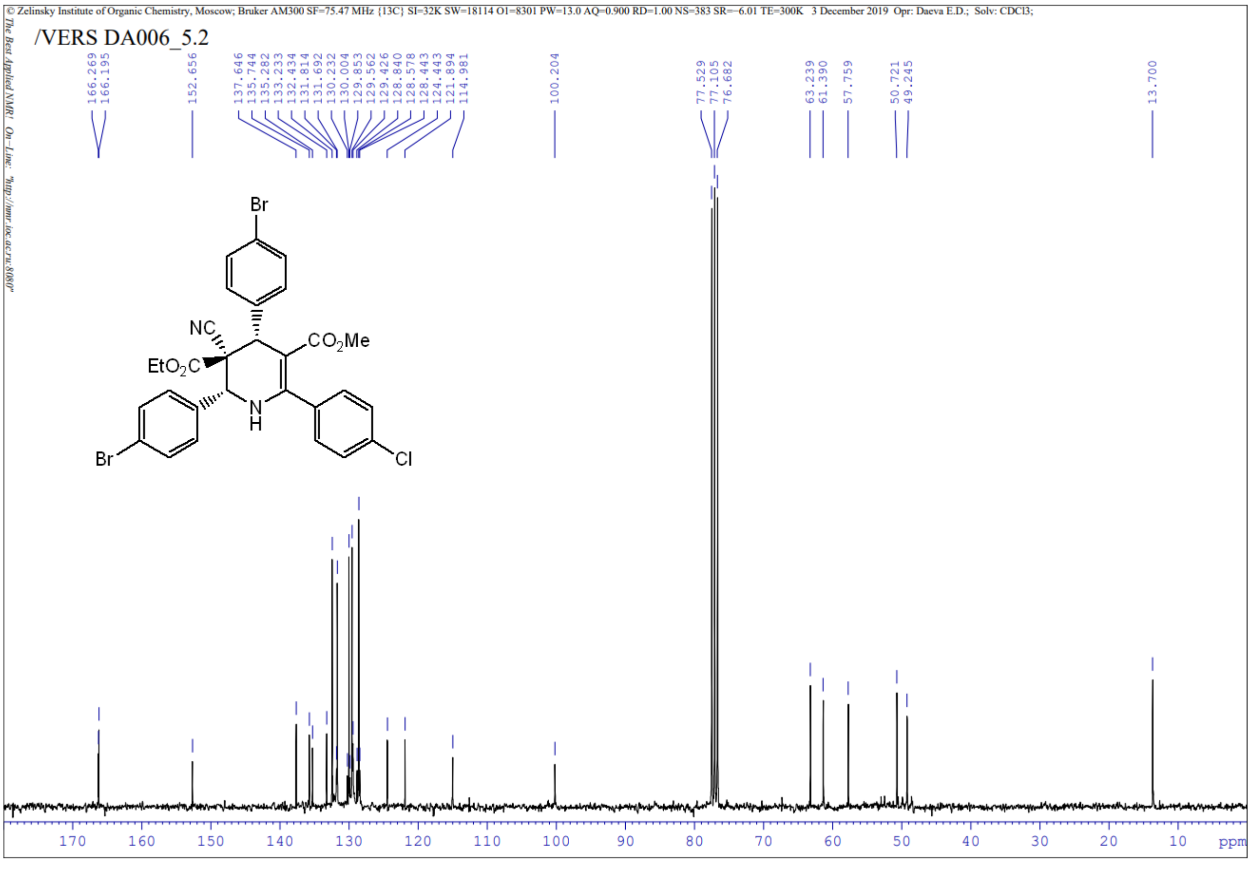

$^{1}$H NMR of ethyl (2*SR*, 3*RS*, 4*SR*, 6*RS*)-5,5-dicyano-2-phenyl-2-hydroxy-4,6-diphenyl-piperidine-3-carboxylate (**6a**)

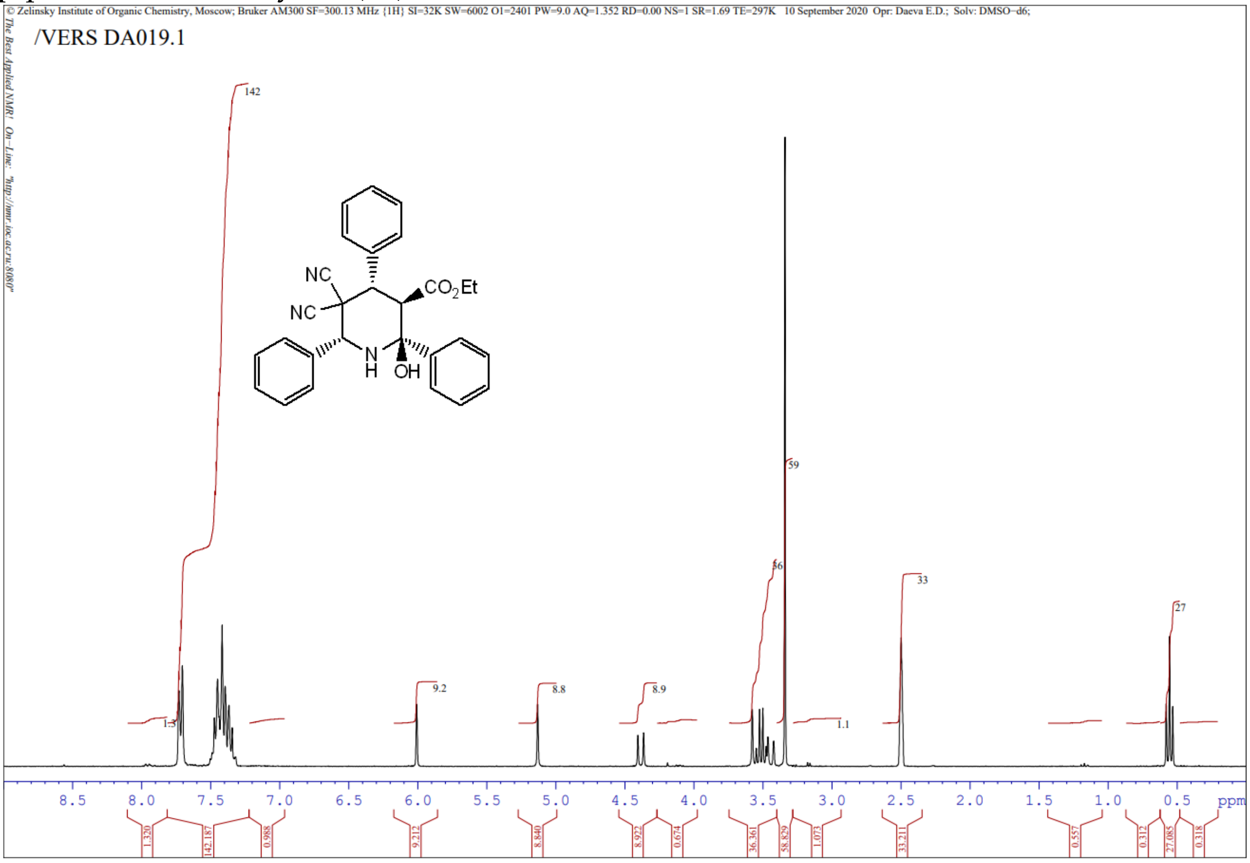


$^{13}$C NMR of **6a**

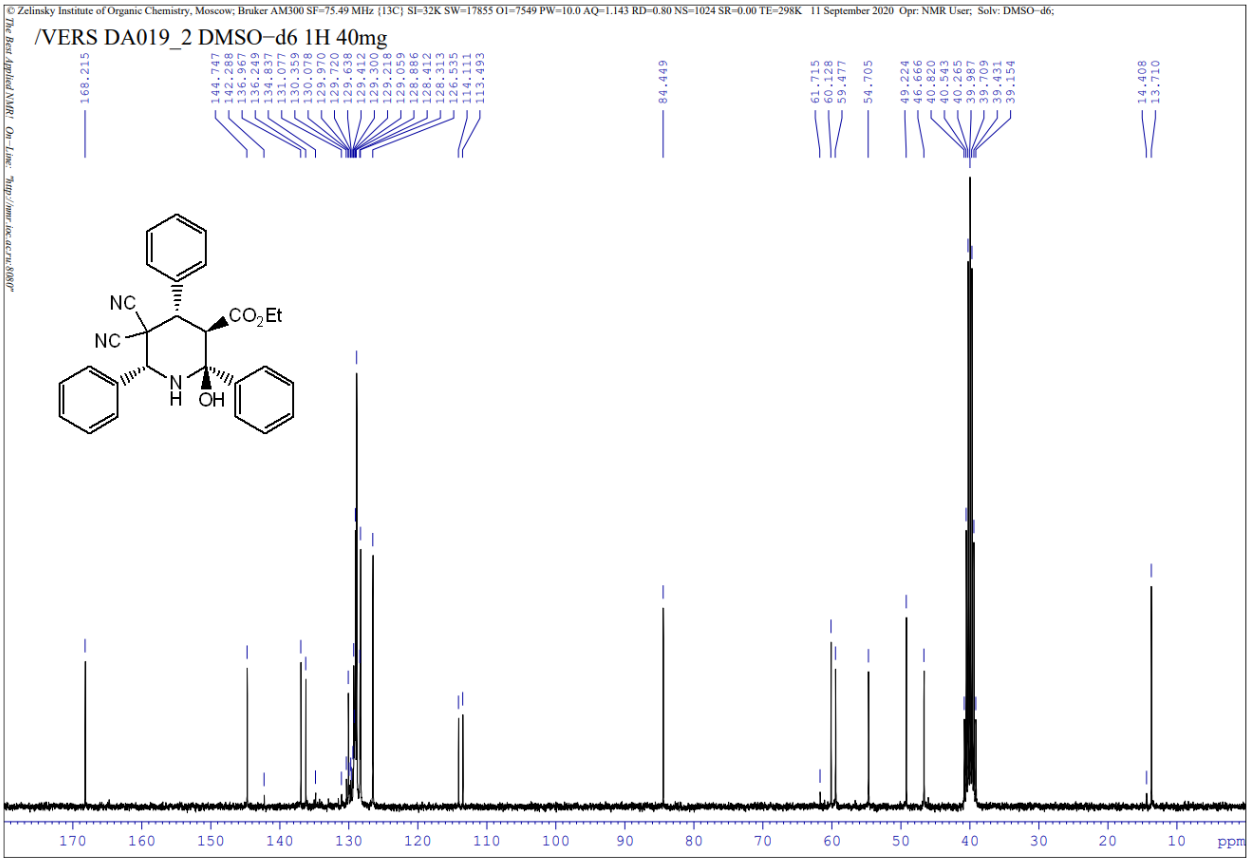

$^{1}$H NMR of ethyl (2*SR*, 3*RS*, 4*SR*, 6*RS*)-5,5-dicyano-2-phenyl-2-hydroxy-4,6-bis(4-methyl)phenyl-piperidine-3-carboxylate (**6b**)

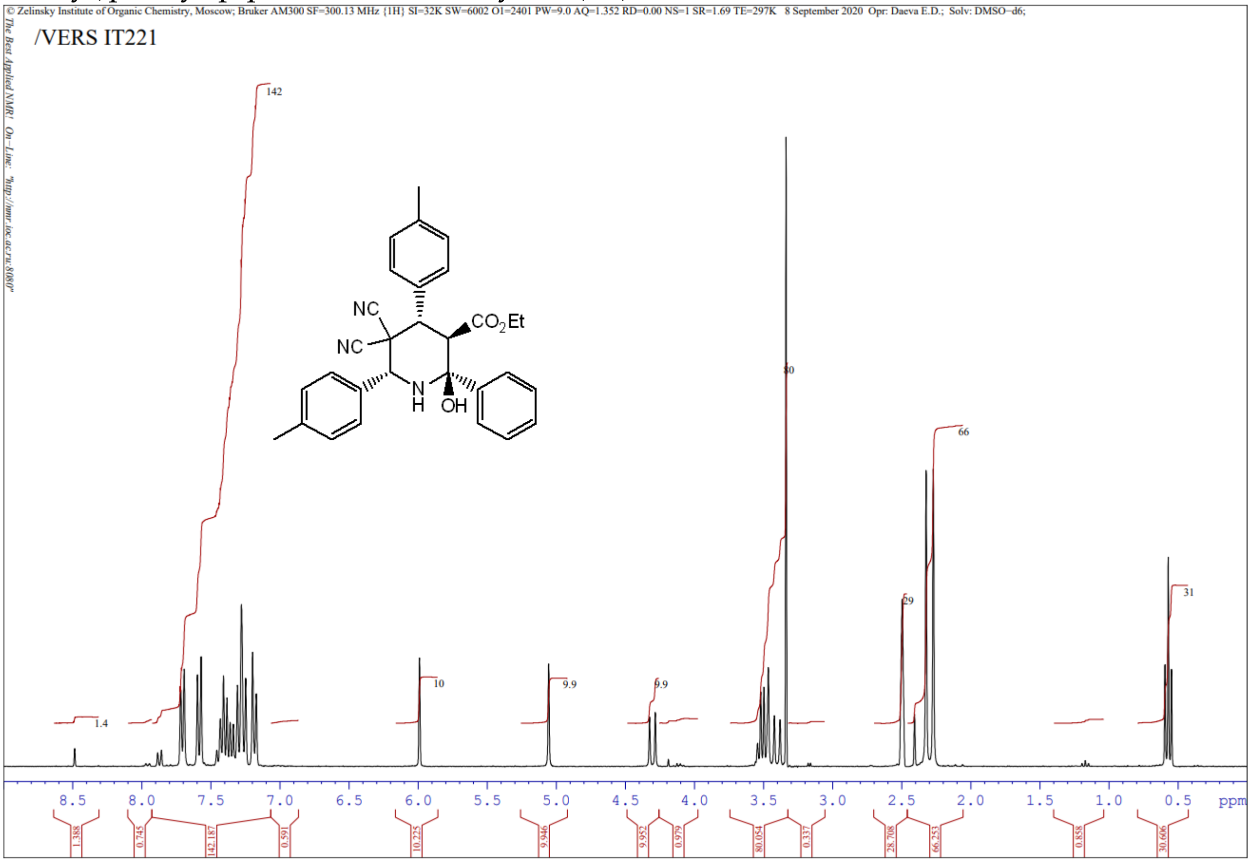


$^{13}$C NMR of **6b**

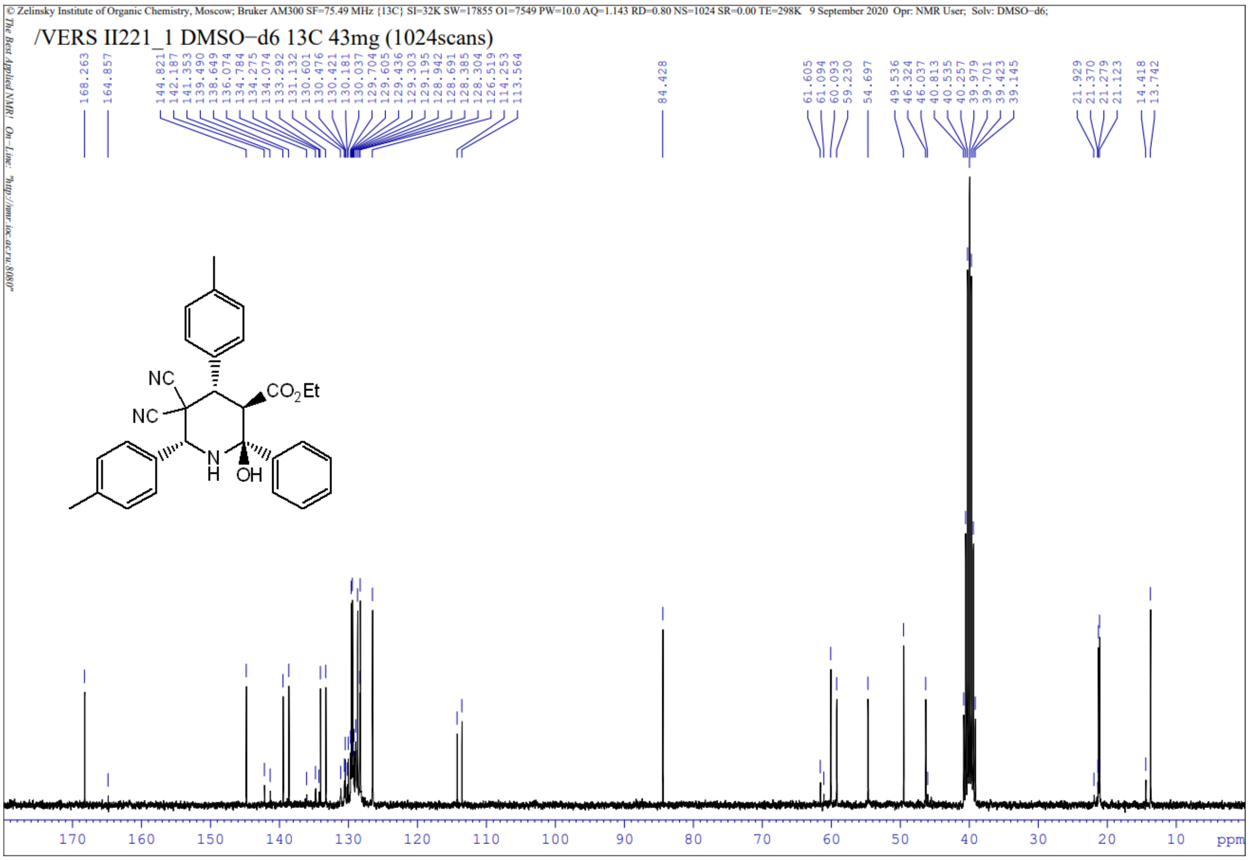

$^{1}$H NMR of ethyl (2*SR*, 3*RS*, 4*SR*, 6*RS*)-5,5-dicyano-2-phenyl-2-hydroxy-4,6-bis(4-chloro)phenyl-piperidine-3-carboxylate (**6c**)

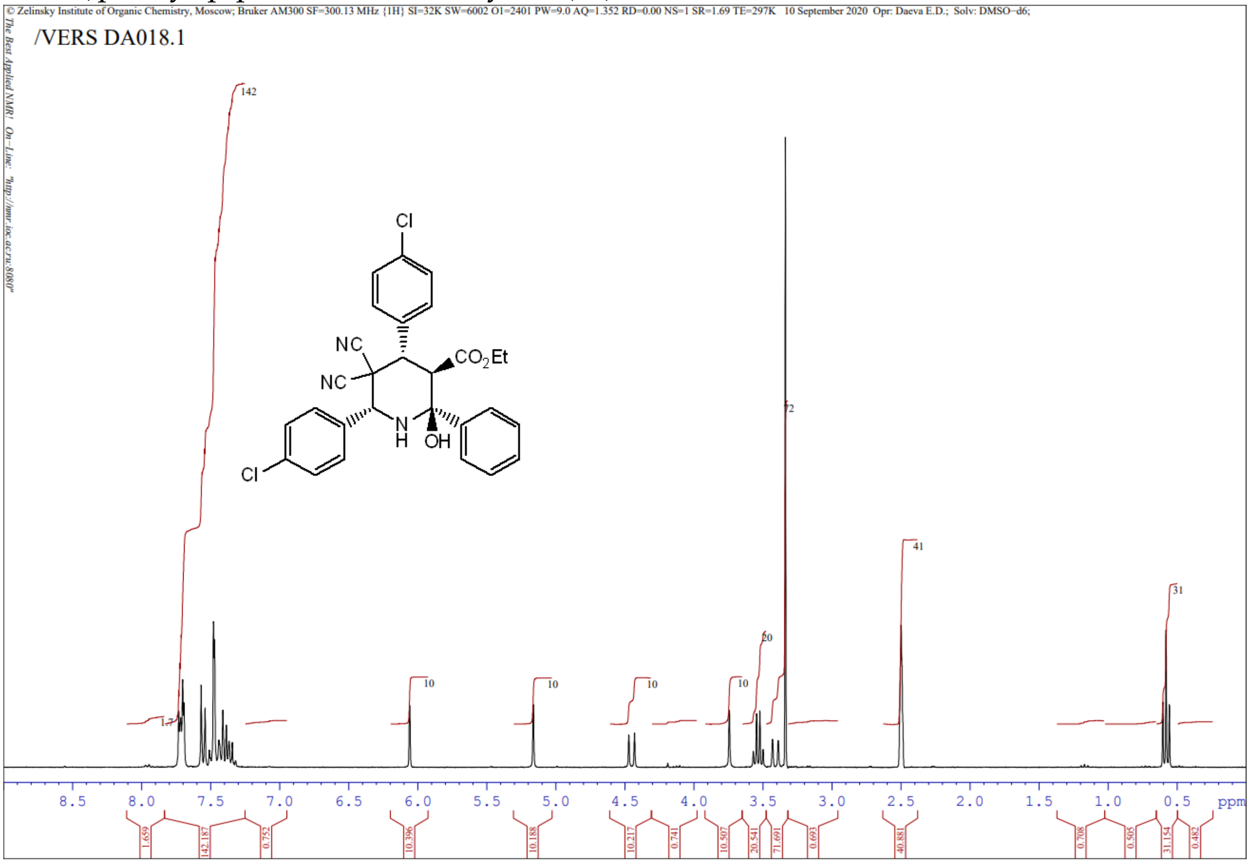


$^{13}$C NMR of **6c**

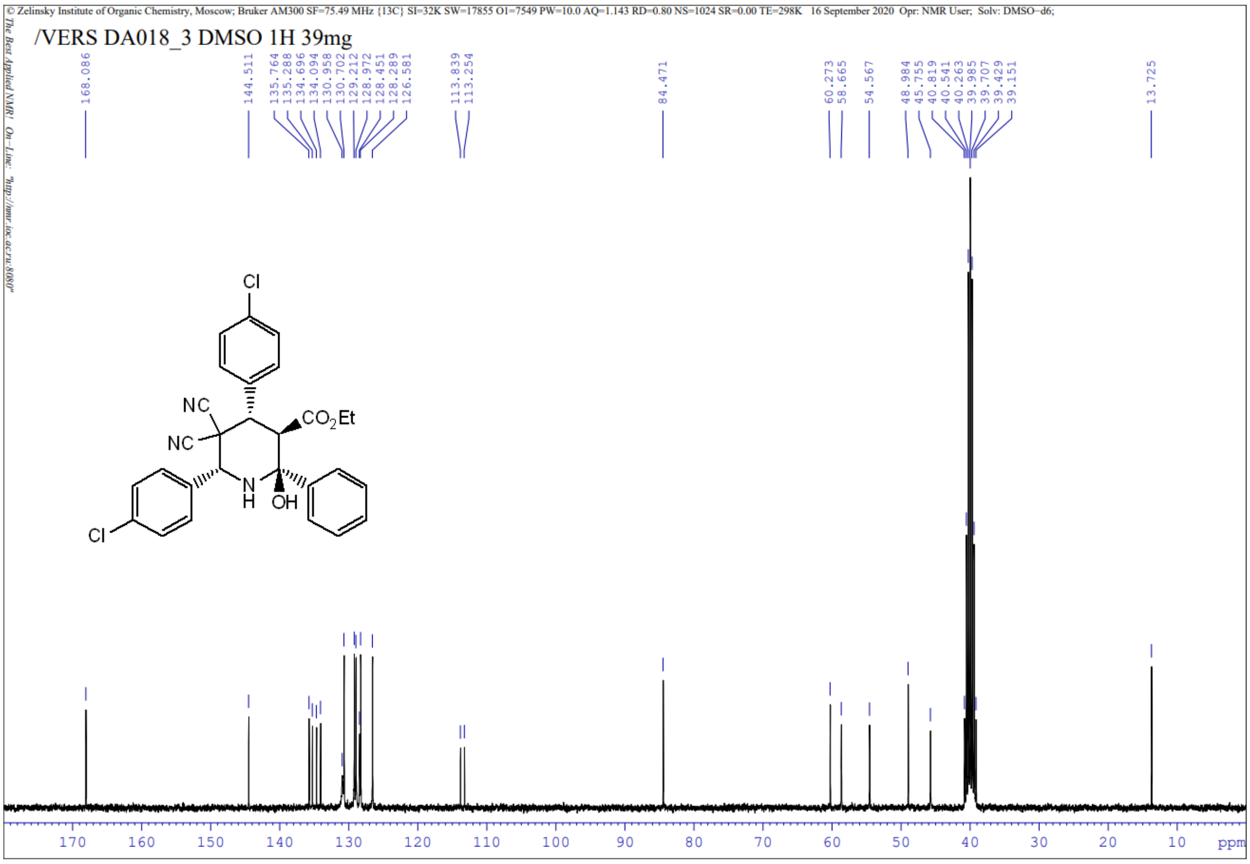

$^{1}$H NMR of methyl (2*SR*, 3*RS*, 4*SR*, 6*RS*)-5,5-dicyano-2-(4-bromo)phenyl-2-hydroxy-4,6-bis(4-methyl)phenyl-piperidine-3-carboxylate (**6d**)

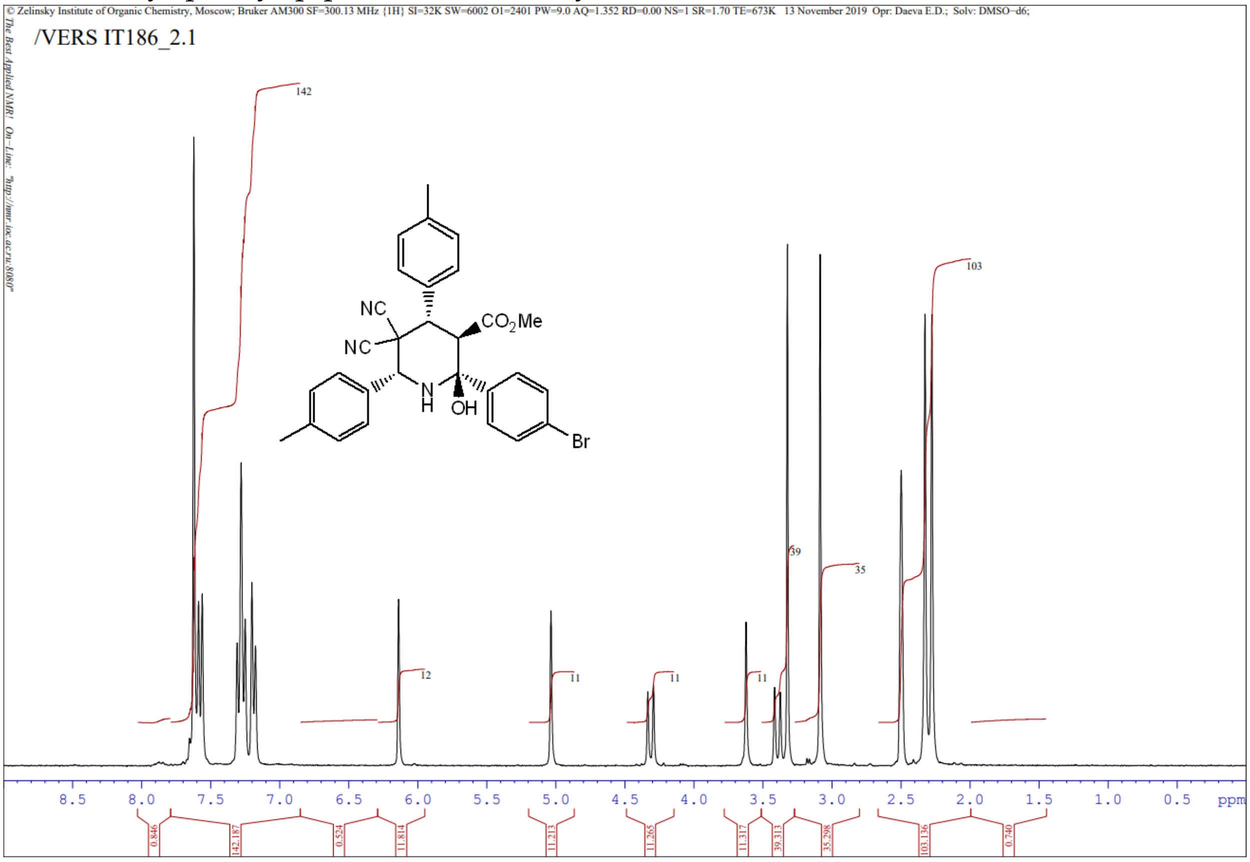

$^{13}$C NMR of **6d**

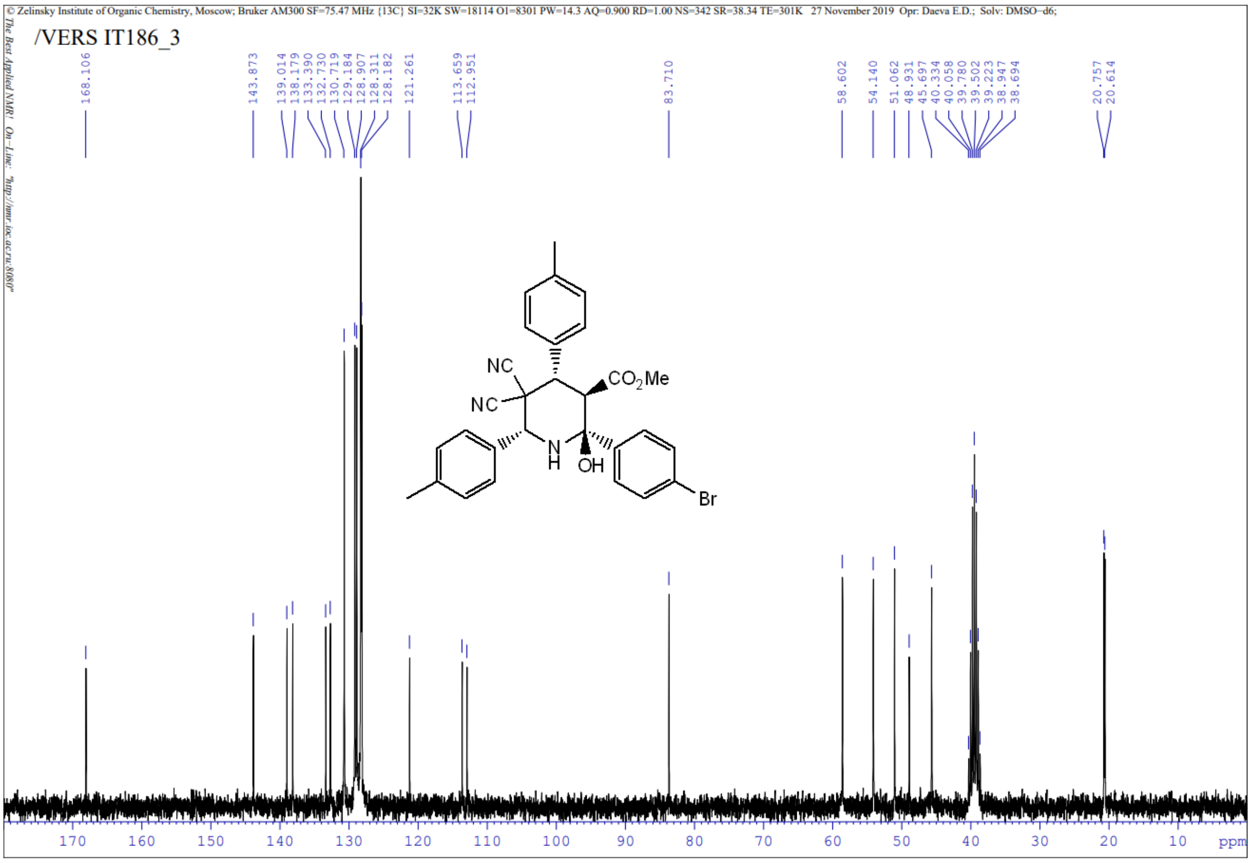

$^{1}$H NMR of methyl(3*RS*, 4*SR*, 6*RS*)-5,5-cyano-2-(4-bromo)phenyl-4,6-bis(4-methyl)phenyl-3,4,5,6-tetrahydropyridine-3-carboxylate (**7**)

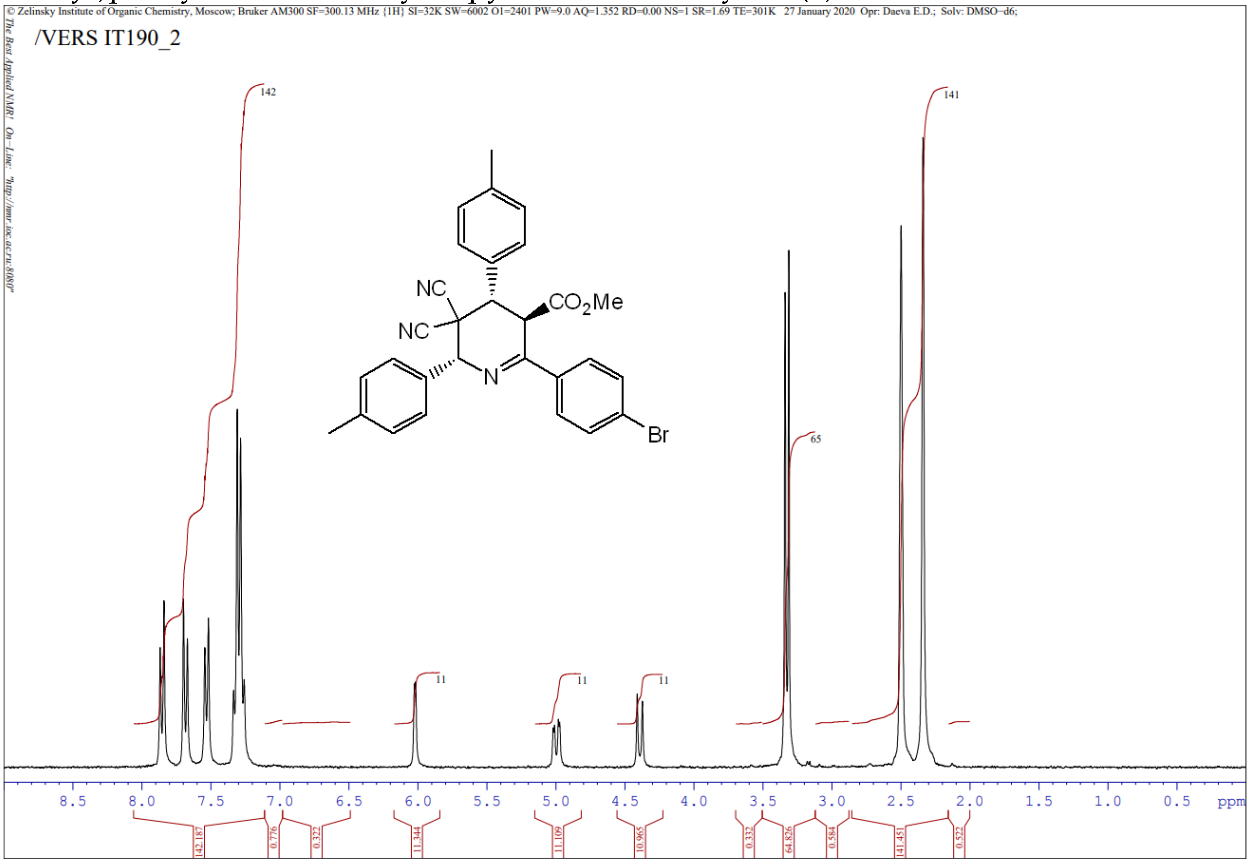


$^{13}$C NMR of **7**

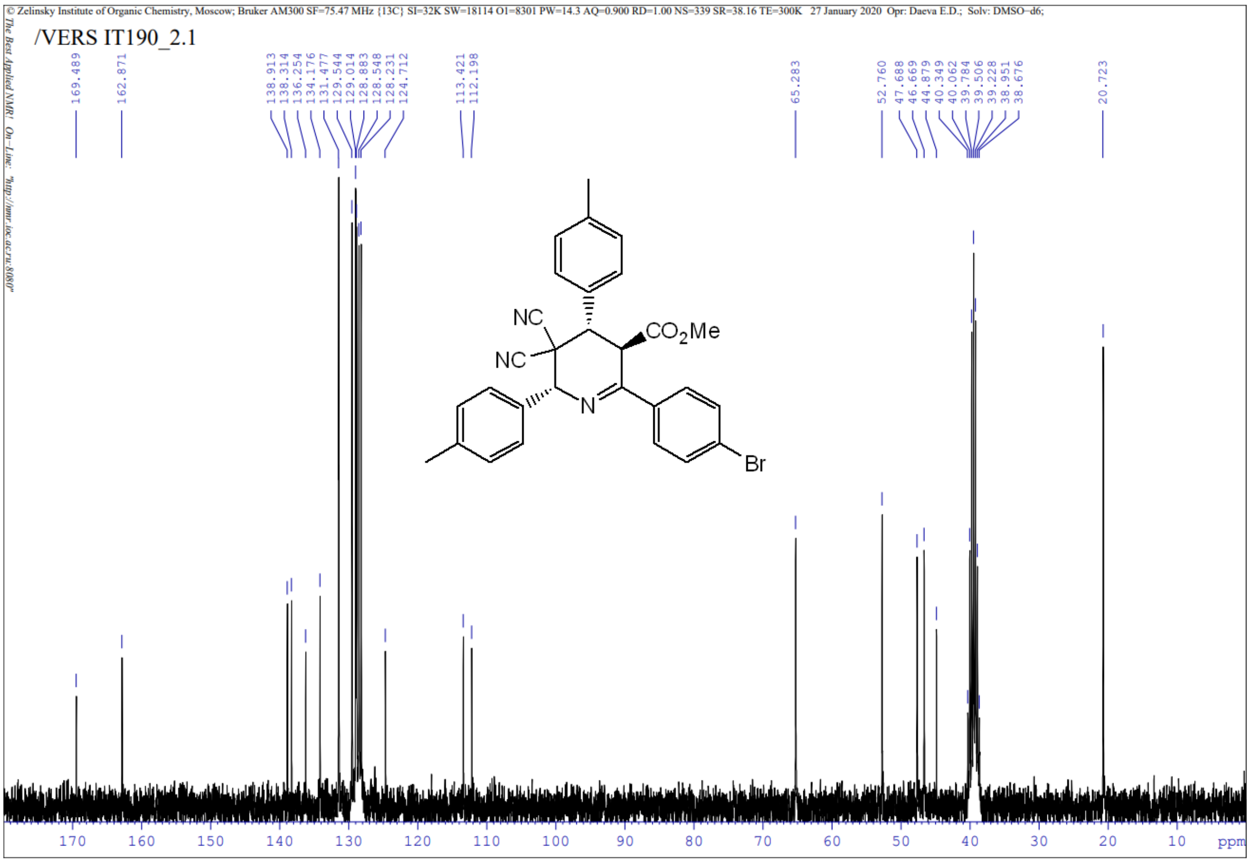

$^{1}$H NMR of 5-ethyl 3-methyl (3*SR*,4*RS*,5*SR*,6*SR*)-6-(4-bromophenyl)-3-cyano-2,4-bis(4-fluorophenyl)- 3,4,5,6-tetrahydropyridine-3,5-dicarboxylate (**8**)

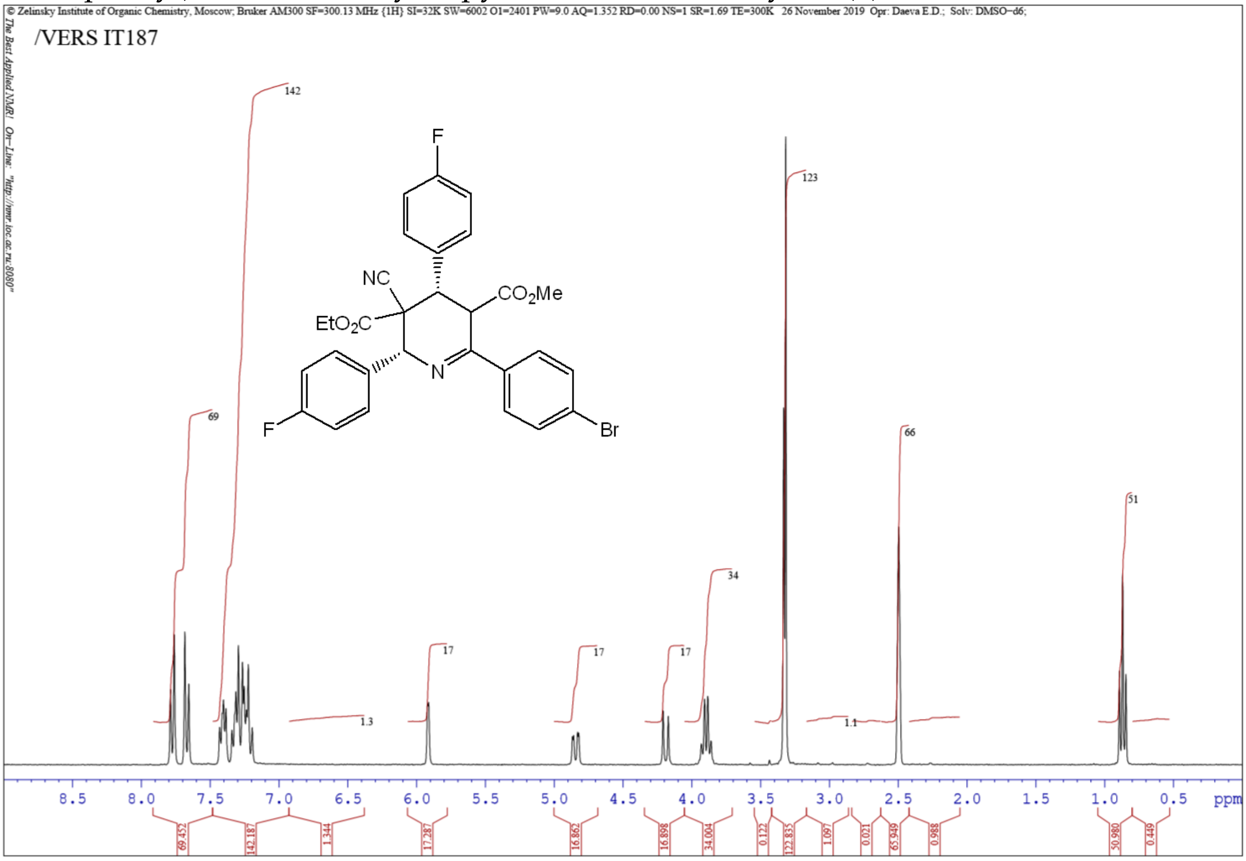


$^{13}$C NMR of **8**

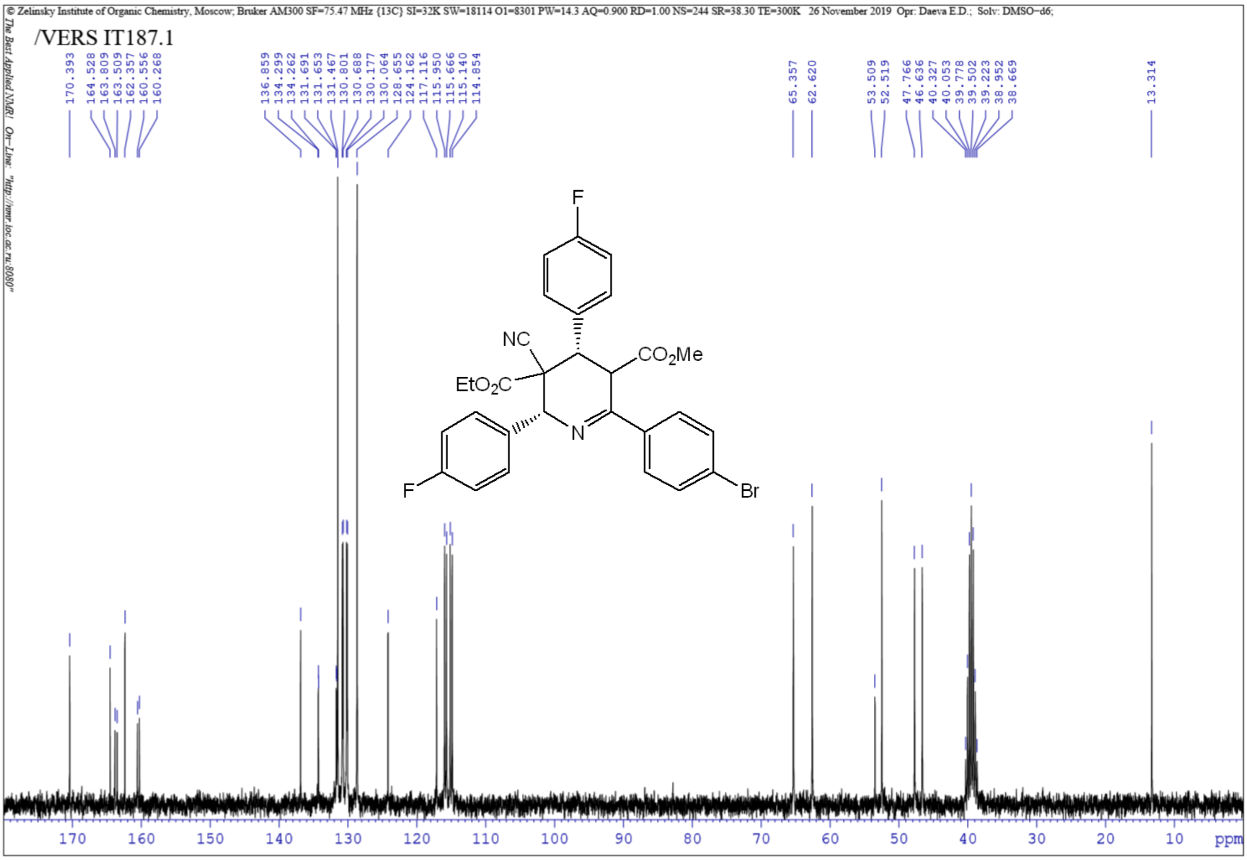

## 2D spectra of compounds 6d, 7

$^{1}$H-$^{1}$H COSY NMR of **6d**

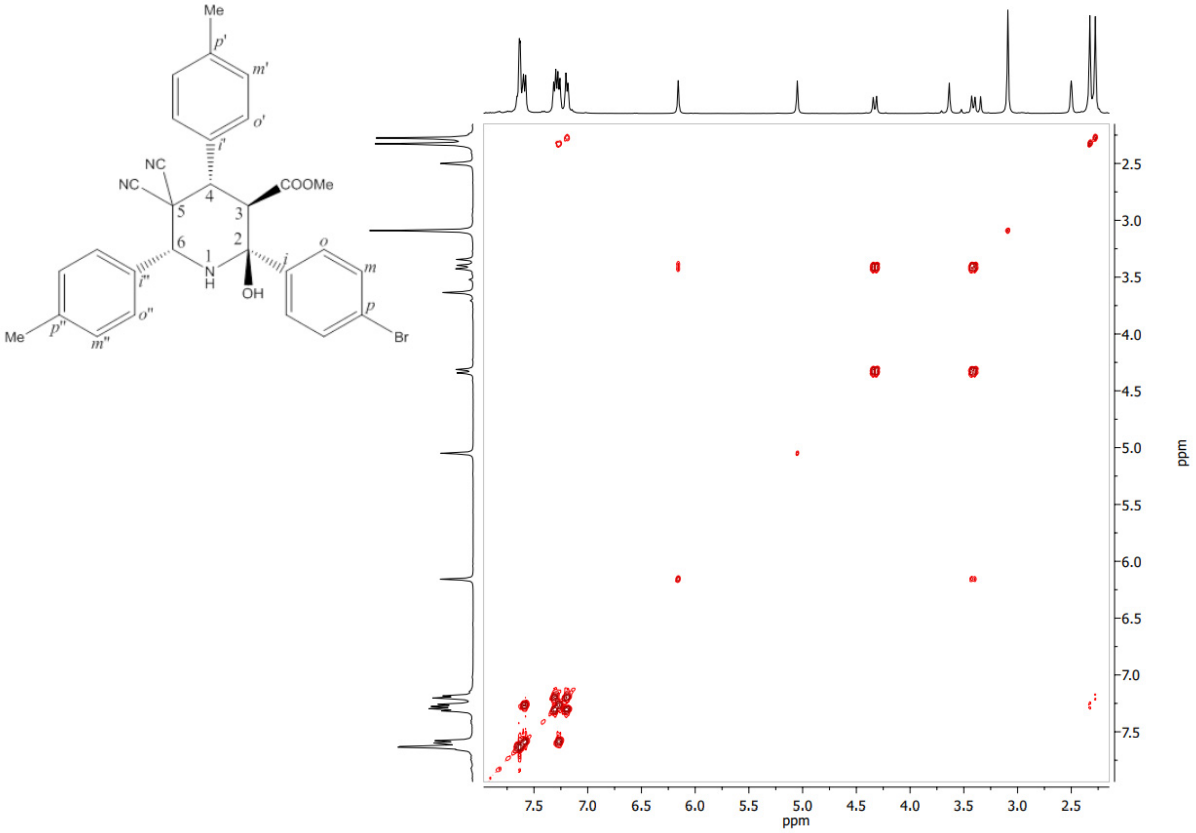

$^{1}$H-$^{13}$C HSQC NMR of **6d**

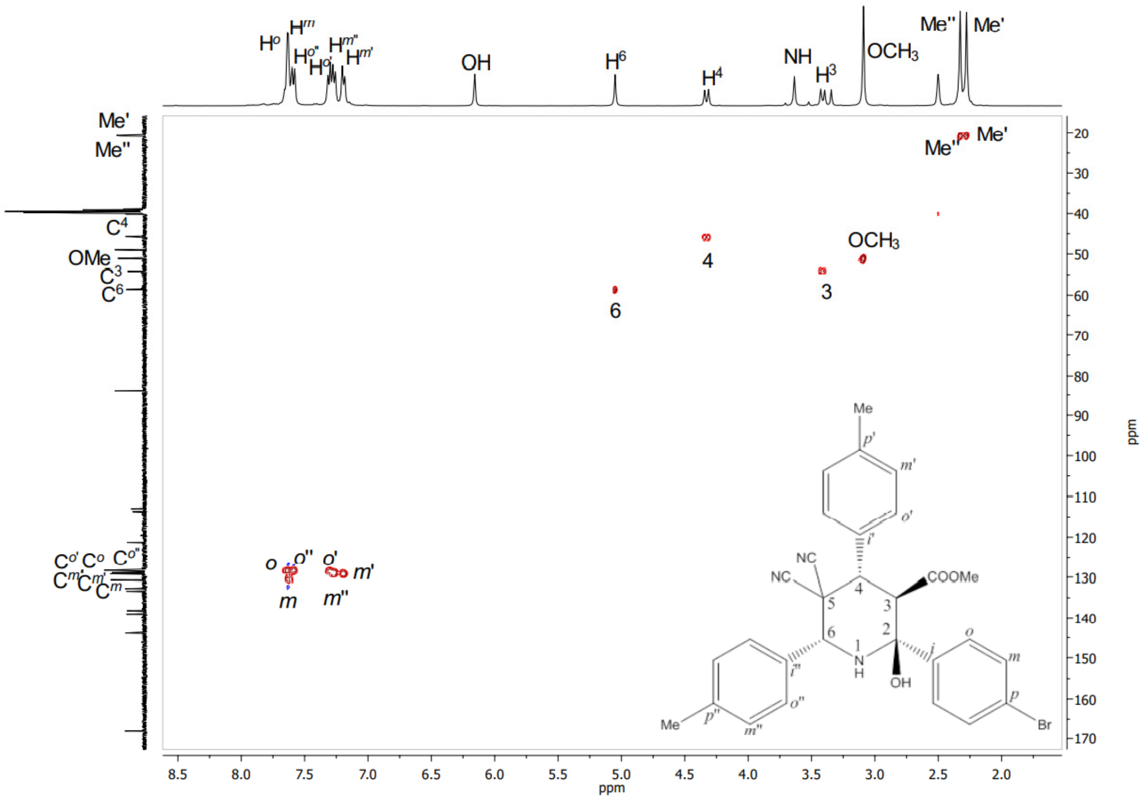

$^{1}H$-$^{13}C$ HMBC NMR of **6d**

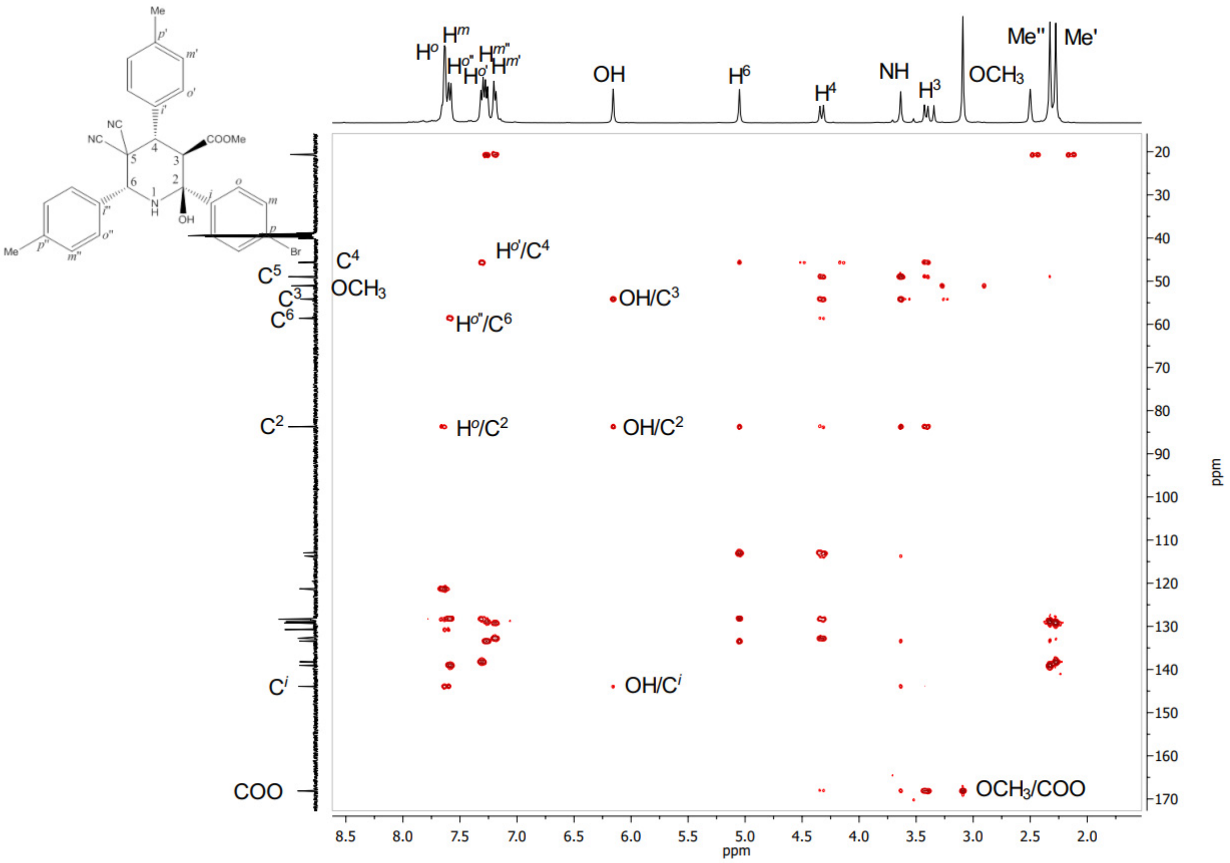


$^{1}H$-$^{1}H$ NOESY of **6d**

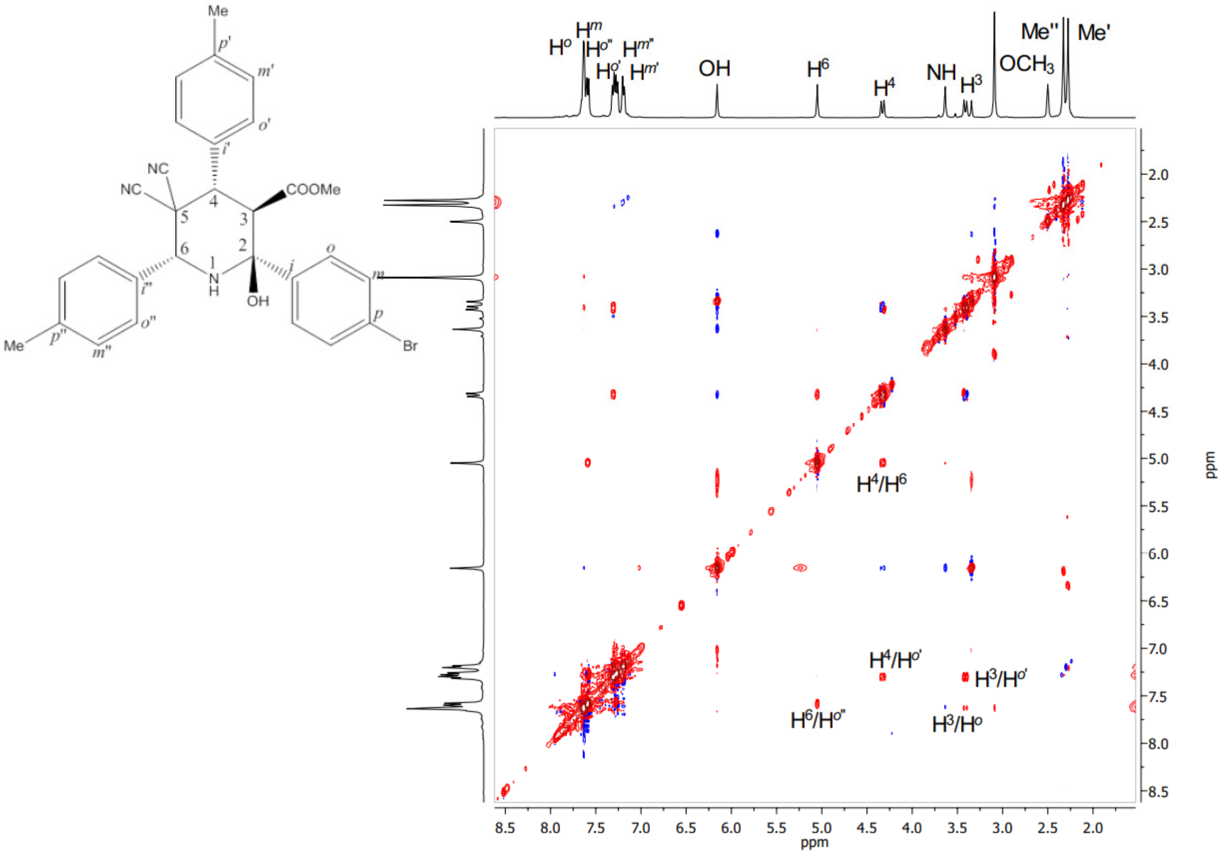

$^{1}$H-$^{1}$H COSY NMR of **7**

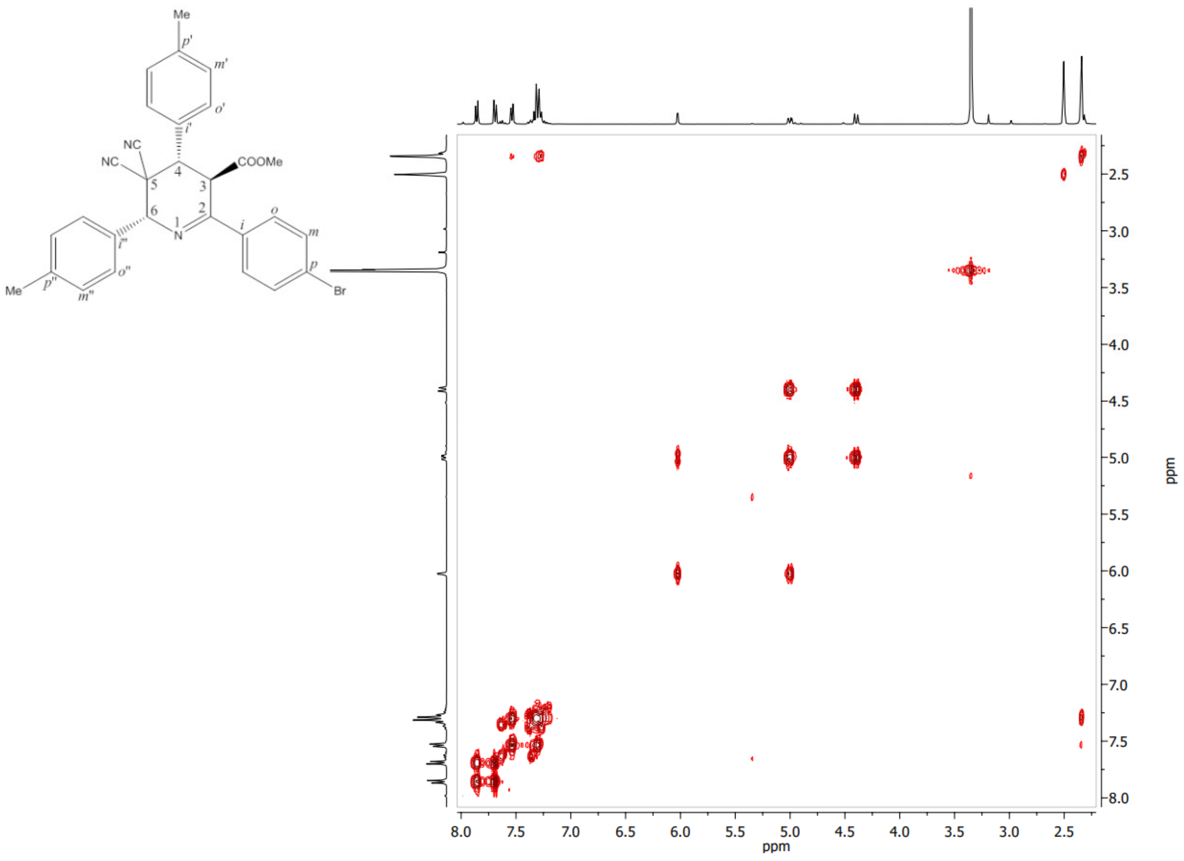

$^{1}$H-$^{13}$C HSQC NMR of **7**

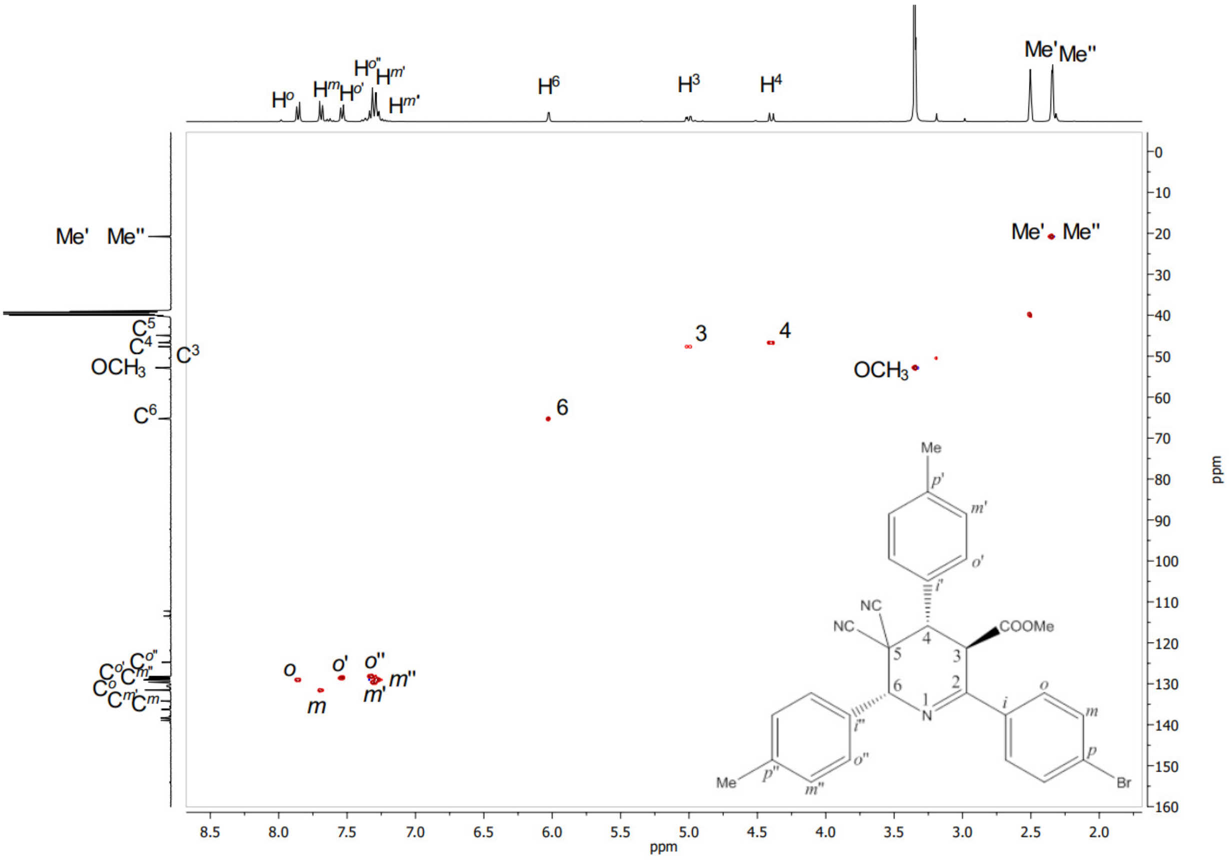

$^1H$-$^1H$ NOESY NMR of **7**

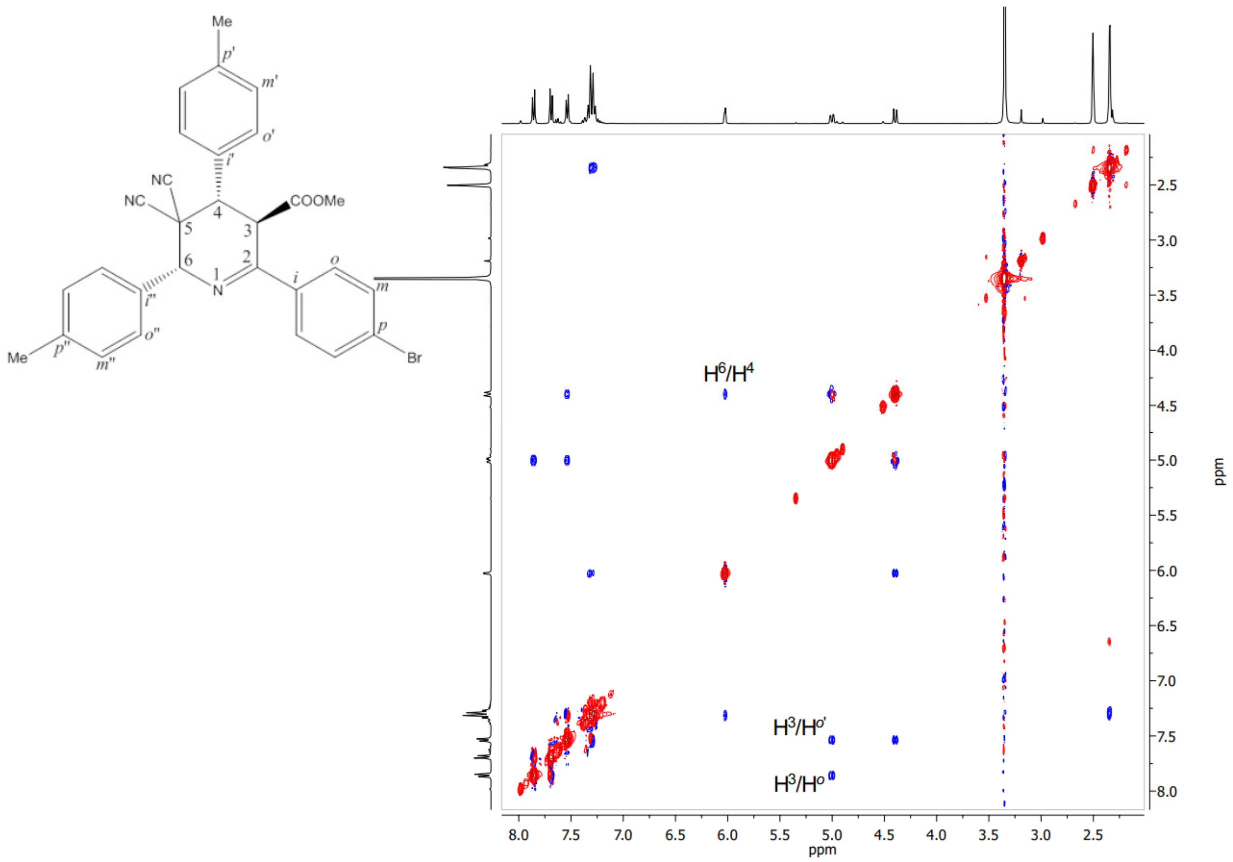

## DFT calculations

DFT calculations were performed with the Gaussian 16 Rev C.01. B3LYP DFT functional with GD3BJ empirical dispersion correction and Def2SVP basis set was used for geometry optimization and calculations of thermodynamics. Data from X-ray diffraction experiment for **7** were used as starting points for geometry optimizations. Cartesian coordinates are given in angstroms; absolute energies for all substances are given in hartrees. Analysis of vibrational frequencies was performed for all optimized structures. All compounds were characterized by only real vibrational frequencies. Wavefunction stability, using stable keyword, was also checked for each molecule

For calculations of optimized geometries, frequencies and thermodynamics following keywords were used:

*# opt freq b3lyp nosymm def2svp empiricaldispersion=gd3bj test*

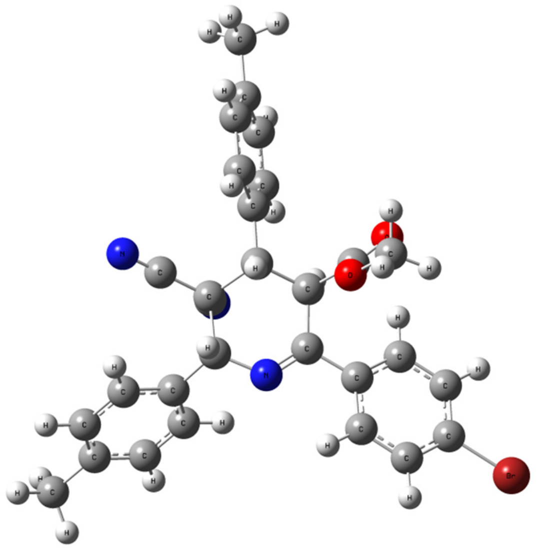

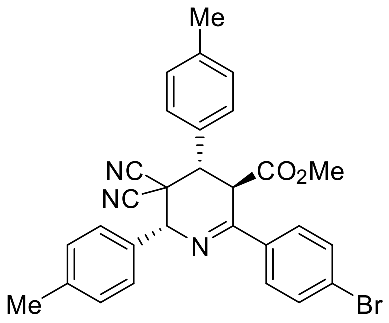


**7**

Charge 0; multiplicity 1

| | | | |
|---|---|---|---|
| Br | -3.02730700 | 5.39387300 | 2.43337100 |
| O | 3.12678900 | 7.51740300 | 4.78284400 |
| O | 3.44796600 | 5.36289600 | 5.34313800 |
| N | 0.90709000 | 4.96470500 | 8.02335100 |
| N | 4.42640300 | 5.75964100 | 11.21921400 |
| N | 1.09800400 | 8.30028600 | 9.88890600 |
| C | 1.11882000 | 5.72374300 | 7.02116100 |
| C | 2.29506500 | 6.69645500 | 6.91139500 |
| H | 1.90904200 | 7.72267300 | 6.98466800 |
| C | 3.34846900 | 6.44862400 | 8.00563100 |
| H | 3.82481300 | 5.48276000 | 7.78706400 |

| | | | |
|---|---|---|---|
| C | 2.61979500 | 6.22253600 | 9.37306600 |
| C | 1.71721000 | 4.92990000 | 9.20995700 |
| H | 2.43150700 | 4.09377200 | 9.10936400 |
| C | 0.14652900 | 5.66566600 | 5.89396100 |
| C | -0.69884700 | 4.54707000 | 5.77892400 |
| H | -0.60234900 | 3.74894200 | 6.51575800 |
| C | -1.63707800 | 4.45813400 | 4.75635700 |
| H | -2.28250700 | 3.58309700 | 4.66883900 |
| C | -1.74730600 | 5.50740300 | 3.83614100 |
| C | -0.93058800 | 6.63430100 | 3.93535700 |
| H | -1.02980800 | 7.45076800 | 3.21922300 |
| C | 0.01441400 | 6.70781900 | 4.96093900 |
| H | 0.64146100 | 7.59712300 | 5.01758300 |
| C | 2.98835800 | 6.60551500 | 5.55632700 |
| C | 4.12074400 | 5.14110400 | 4.10090500 |
| H | 4.41133500 | 4.08417000 | 4.09645900 |
| H | 5.00772000 | 5.78673900 | 4.02378900 |
| H | 3.44937200 | 5.35989000 | 3.25780800 |
| C | 4.43397100 | 7.50073300 | 8.04461800 |
| C | 4.14577300 | 8.87409500 | 8.07011300 |
| H | 3.11231200 | 9.22315900 | 8.05674700 |
| C | 5.17345700 | 9.81248100 | 8.12295400 |
| H | 4.92393700 | 10.87671200 | 8.14401000 |
| C | 6.52132100 | 9.41876100 | 8.14693700 |
| C | 6.80304300 | 8.04726500 | 8.11790500 |
| H | 7.84249500 | 7.71000300 | 8.13691200 |
| C | 5.77557800 | 7.10345700 | 8.06894000 |
| H | 6.02148200 | 6.03854700 | 8.05576100 |
| C | 7.61992500 | 10.44845800 | 8.20463200 |
| H | 7.54564000 | 11.05548900 | 9.12207700 |
| H | 7.55815100 | 11.14466800 | 7.35214200 |
| H | 8.61470400 | 9.98040500 | 8.18933900 |
| C | 3.61720900 | 5.99776600 | 10.42661200 |
| C | 1.78209900 | 7.38470100 | 9.70675200 |
| C | 0.86759200 | 4.67724900 | 10.44087000 |
| C | 1.39187900 | 3.98426600 | 11.53702800 |
| H | 2.41038800 | 3.59135500 | 11.49841900 |
| C | 0.62403300 | 3.79249500 | 12.68758000 |
| H | 1.05143200 | 3.24678000 | 13.53269600 |
| C | -0.68507100 | 4.28425100 | 12.77398100 |
| C | -1.20505400 | 4.97042400 | 11.66439300 |
| H | -2.22657100 | 5.35870100 | 11.70311900 |
| C | -0.44383600 | 5.16434000 | 10.51408400 |
| H | -0.86782200 | 5.69080300 | 9.65784100 |

| | | | |
|---|---|---|---|
| C | -1.51479400 | 4.09624500 | 14.01820600 |
| H | -2.50612100 | 3.67836200 | 13.77958100 |
| H | -1.02352600 | 3.42170400 | 14.73439500 |
| H | -1.68561600 | 5.05959400 | 14.52793000 |

| DFT B3LYP/Def2SVP, gas phase | |
|---|---|
| Total electronic energy= | -4007.193039 $E_0$ |
| Sum of electronic and zero-point Energies= | -4006.731807 $E_0 + E_{ZPE}$ |
| Sum of electronic and thermal Energies= | -4006.698980 $E_0 + E_{tot}$ |
| Sum of electronic and thermal Enthalpies= | -4006.698036 $E_0 + H_{corr}$ |
| Sum of electronic and thermal Free Energies= | -4006.802574 $E_0 + G_{corr}$ |
| Zero-point correction *(unscaled)* = | 0.461232 |
| Number of imaginary vibrational frequencies = 0 | |

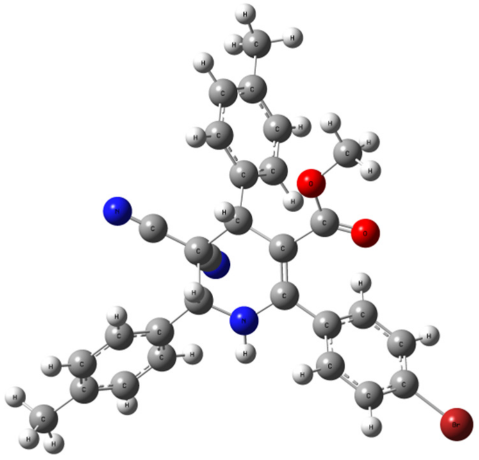

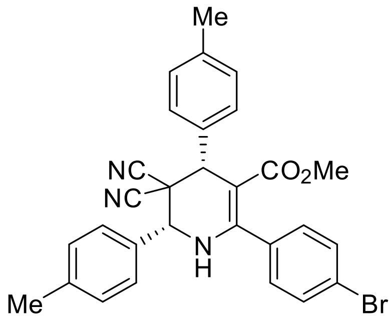


**4t**

Charge 0; multiplicity 1

| | | | |
|---|---|---|---|
| Br | -3.31998100 | 5.91938200 | 2.66104300 |
| O | 2.60415000 | 6.24639500 | 4.36735500 |
| O | 4.45487100 | 5.68744100 | 5.50582400 |
| N | 0.67249500 | 5.15100100 | 8.09694300 |
| N | 4.23239600 | 5.44226500 | 11.29522100 |
| N | 1.35506100 | 8.27446700 | 9.59563600 |
| C | 1.17056200 | 5.58436300 | 6.89658400 |
| C | 2.50703800 | 5.86887500 | 6.75732000 |
| C | 3.43893400 | 5.95638900 | 7.95778300 |
| H | 4.05308000 | 5.04514500 | 8.02198800 |
| C | 2.61643800 | 5.98963200 | 9.30206000 |
| C | 1.51957600 | 4.85217800 | 9.22064000 |

| | | | |
|---|---|---|---|
| H | 2.09678900 | 3.93257600 | 9.02201400 |
| C | 0.13654200 | 5.65913000 | 5.83246400 |
| C | -0.72791700 | 4.57213600 | 5.62835500 |
| H | -0.58667000 | 3.65436900 | 6.20350400 |
| C | -1.75125800 | 4.63668500 | 4.68154500 |
| H | -2.40938200 | 3.78318600 | 4.51458600 |
| C | -1.92074300 | 5.81184500 | 3.94732400 |
| C | -1.08034900 | 6.91205500 | 4.14462000 |
| H | -1.22787500 | 7.82486000 | 3.56651500 |
| C | -0.05319000 | 6.82837000 | 5.08095000 |
| H | 0.60998900 | 7.67842300 | 5.23441100 |
| C | 3.13352800 | 5.97446600 | 5.42345700 |
| C | 5.21182400 | 5.85312500 | 4.31321600 |
| H | 6.25201100 | 5.62784000 | 4.57879900 |
| H | 5.12968500 | 6.88584600 | 3.94160800 |
| H | 4.85972300 | 5.17141200 | 3.52432000 |
| C | 4.39559900 | 7.13839000 | 7.89873300 |
| C | 3.99107000 | 8.38374700 | 7.40093600 |
| H | 2.97426600 | 8.51233200 | 7.02601100 |
| C | 4.87644500 | 9.46024100 | 7.37467900 |
| H | 4.53830100 | 10.42164200 | 6.97850900 |
| C | 6.19169500 | 9.33217100 | 7.84694400 |
| C | 6.58970800 | 8.08261000 | 8.34212300 |
| H | 7.61036700 | 7.94981300 | 8.71077800 |
| C | 5.70634900 | 7.00251100 | 8.36739100 |
| H | 6.04430300 | 6.03761300 | 8.75271000 |
| C | 7.13331200 | 10.50922700 | 7.84314100 |
| H | 6.94386400 | 11.16801500 | 8.70838400 |
| H | 7.00921900 | 11.12229100 | 6.93693900 |
| H | 8.18381100 | 10.18728500 | 7.89893100 |
| C | 3.51618000 | 5.72409600 | 10.43084200 |
| C | 1.94279800 | 7.28379100 | 9.48627900 |
| C | 0.73288600 | 4.66565200 | 10.49497500 |
| C | 1.08822500 | 3.64283200 | 11.38436700 |
| H | 1.91837600 | 2.97681800 | 11.13730400 |
| C | 0.39840900 | 3.47028800 | 12.58238500 |
| H | 0.69324400 | 2.66471700 | 13.25995800 |
| C | -0.66922500 | 4.31205100 | 12.93163700 |
| C | -1.02083900 | 5.33159200 | 12.03622700 |
| H | -1.84672800 | 6.00342600 | 12.28317100 |
| C | -0.33018800 | 5.51284300 | 10.83694300 |
| H | -0.61598000 | 6.33490300 | 10.17799700 |
| C | -1.39314800 | 4.13539200 | 14.24118100 |
| H | -1.58396700 | 3.07218300 | 14.45493900 |

| | | | |
|---|---|---|---|
| H | -0.79117900 | 4.53095000 | 15.07717300 |
| H | -2.35629700 | 4.66600100 | 14.24624600 |
| H | -0.32804900 | 5.17872000 | 8.24374000 |

| DFT B3LYP/Def2SVP, gas phase | | |
|---|---|---|
| Total electronic energy= | -4007.198532 | $E_0$ |
| Sum of electronic and zero-point Energies= | -4006.737455 | $E_0 + E_{ZPE}$ |
| Sum of electronic and thermal Energies= | -4006.704405 | $E_0 + E_{tot}$ |
| Sum of electronic and thermal Enthalpies= | -4006.70346 | $E_0 + H_{corr}$ |
| Sum of electronic and thermal Free Energies= | -4006.808530 | $E_0 + G_{corr}$ |
| Zero-point correction *(unscaled)* = | 0.461077 | |
| Number of imaginary vibrational frequencies = 0 | | |
